# Dimers of D76N-β_2_-microglobulin display potent antiamyloid aggregation activity

**DOI:** 10.1016/j.jbc.2022.102659

**Published:** 2022-10-31

**Authors:** Roberto Maya-Martinez, Yong Xu, Nicolas Guthertz, Martin Walko, Theodoros K. Karamanos, Frank Sobott, Alexander L. Breeze, Sheena E. Radford

**Affiliations:** 1 Astbury Centre for Structural Molecular Biology, School of Molecular and Cellular Biology, Faculty of Biological Sciences, University of Leeds, Leeds, United Kingdom; 2 Astbury Centre for Structural Molecular Biology, School of Chemistry, University of Leeds, Leeds, United Kingdom

**Keywords:** amyloid, D76N-β_2_m, NMR, cross-linking, amyloid inhibitor, oligomer, β_2_m, β_2_-microglobulin, ESI-MS, electrospray ionization mass spectrometry, MS, mass spectrometry, MTSL, S-(1-oxyl-2,2,5,5-tetramethyl-2,5-dihydro-1H-pyrrol-3-yl) methyl methanesulfonothioate, PRE, paramagnetic relaxation enhancement, ThT, thioflavin T, XL-D, crosslinked dimer

## Abstract

Self-association of WT β_2-_microglobulin (WT-β_2_m) into amyloid fibrils is associated with the disorder dialysis related amyloidosis. In the familial variant D76N-β_2_m, the single amino acid substitution enhances the aggregation propensity of the protein dramatically and gives rise to a disorder that is independent of renal dysfunction. Numerous biophysical and structural studies on WT- and D76N-β_2_m have been performed in order to better understand the structure and dynamics of the native proteins and their different potentials to aggregate into amyloid. However, the structural properties of transient D76N-β_2_m oligomers and their role(s) in assembly remained uncharted. Here, we have utilized NMR methods, combined with photo-induced crosslinking, to detect, trap, and structurally characterize transient dimers of D76N-β_2_m. We show that the crosslinked D76N-β_2_m dimers have different structures from those previously characterized for the on-pathway dimers of ΔN6-β_2_m and are unable to assemble into amyloid. Instead, the crosslinked D76N-β_2_m dimers are potent inhibitors of amyloid formation, preventing primary nucleation and elongation/secondary nucleation when added in substoichiometric amounts with D76N-β_2_m monomers. The results highlight the specificity of early protein–protein interactions in amyloid formation and show how mapping these interfaces can inform new strategies to inhibit amyloid assembly.

β_2_-microglobulin (β_2_m) is a 99-residue protein that adopts a β-sandwich immunoglobulin (Ig) fold (1). The biological function of β_2_m is to chaperone and support the assembly of the MHC-I that is required to present epitopes to the immune system (2, 3). During its recycling process, the heavy chain of MHC-1 (bound to the cell membrane) is internalized by the host cell, whereas β_2_m monomers are released into the serum and subsequently eliminated through degradation by the kidneys (4). Under normal physiological conditions, the concentration of β_2_m in the serum is between 0.6 to 1.8 mg/l (5). However, in individuals suffering from renal dysfunction the concentration of β_2_m in the serum can increase up to 60-fold (6). As a result, progressive deposition of β_2_m into amyloid fibrils occurs, which accumulate mainly in collagenous joints and leads to the disorder known as dialysis-related amyloidosis (7). A wealth of previous studies has shown how truncation of the N-terminal six amino acids of WT-β_2_m (creating the protein ΔN6-β_2_m) (8) and/or specific interactions with collagen (9, 10, 11), glycosaminoglycans (12) or other cellular factors (13) are crucial for the self-assembly of WT-β_2_m into amyloid and the development of dialysis-related amyloidosis.

The first example of a familial mutation resulting in β_2_m amyloidosis was reported a decade ago and involves substitution of the highly conserved Asp76 with Asn (14). This variant has a dramatic effect on the properties of the protein, despite its location in a solvent-exposed loop (Fig. 1*A*). The D76N-β_2_m variant retains a native Ig fold (RMSD compared with WT-β_2_m of 0.59 Å (C_α_ atoms) (14)) is less stable than WT-β_2_m (ΔΔG° = 8.36 kJ/mol at pH 7.4 (15, 16)) and aggregates more rapidly than the normally highly aggregation-resistant WT protein *in vitro* and *in vivo* (14, 15, 17). The variant also changes the phenotype of the disease, resulting in the deposition of D76N-β_2_m amyloid fibrils in the liver, spleen, salivary glands, and heart without renal dysfunction (14). Individuals expressing D76N-β_2_m have normal kidney function and the concentration of β_2_m in serum remains within the normal physiological range.

**Figure 1:**
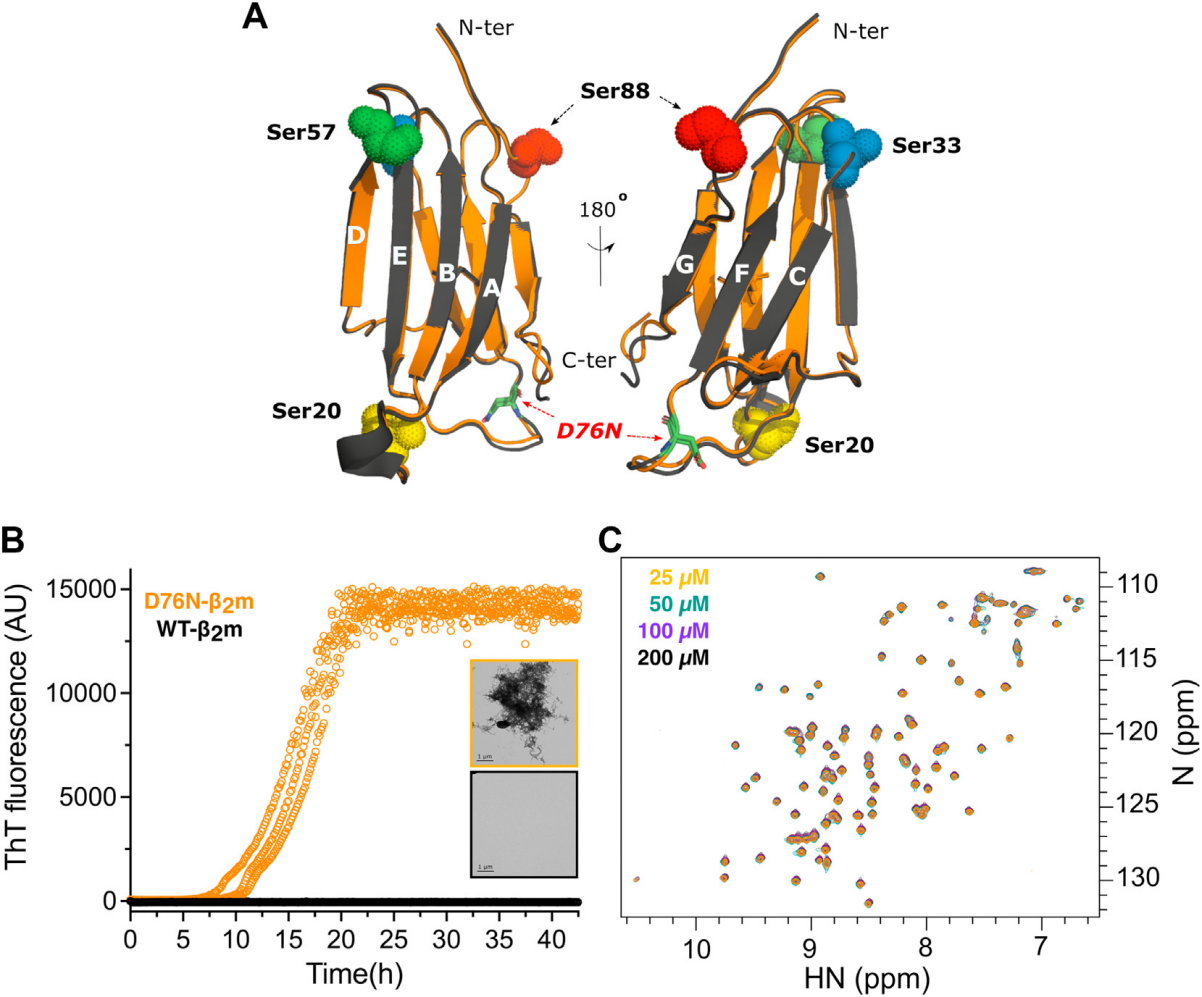
**Structure and aggregation potential of D76N-β**_**2**_**m.***A*,. superposition of the 3D structures of WT- (*gray*, PDB: 2YXF) (37) and D76N-β_2_m (*light brown*, PDB: 4FXL) (14). Residue 76 is located in the EF loop (highlighted in *green stick*). Residues Ser20, Ser33, Ser57, and Ser88, used as PRE probes when mutated to Cys, are shown in spheres (*yellow*, *blue*, *green*, and *red*, respectively). *B*. ThT fibrillation assay of D76N-β_2_m (*orange*) and WT-β_2_m (*black*). The experiment was performed at 37 °C, 600 rpm shaking in 25 mM sodium phosphate, 115 mM of NaCl, 10 μM ThT, pH 6.2, using a protein concentration of 20 μM. Three replicates are shown for each protein. Inset: negative stain electron micrographs of the material at the endpoint of the reaction (each condition is outlined using the same color as the ThT curve). The scale bar represents 1 μm. *C*, 2D ^HN^HSQC NMR spectra of ^15N^D76N-β_2_m at protein concentrations of 25 μM, 50 μM, 100 μM, and 200 μM (*orange*, *green*, *purple*, and *black*, respectively). Spectra were acquired at 750 MHz, in the fibrillation buffer, without ThT. β_2_m, β_2_-microglobulin; PDB, Protein Data Bank; ThT, thioflavin T.

To better understand the aggregation mechanism of D76N-β_2_m, a series of biophysical investigations have been carried out (15, 17, 18, 19, 20, 21). The self-assembly mechanisms of WT-β_2_m and ΔN6-β_2_m have been investigated previously and shown to be initiated by partial unfolding to form an intermediate in which the native *cis* Pro32 isomerizes to the *trans* form, known as I_T_ (22, 23, 24, 25). The structure of I_T_ is mimicked by ΔN6-β_2_m, which also retains its all antiparallel β-stranded Ig structure, has a *trans* Pro32, a repacked hydrophobic core and destabilization of the hydrogen bonds across its Ig fold (26). The rate of aggregation of WT-β_2_m is directly correlated with the concentration of I_T_, demonstrating the intermediate to be on-pathway to amyloid (25). Assembly of ΔN6 thereafter involves the formation of native-like dimers and hexamers, which accumulate before the transition to the all-parallel structure of their amyloid fold (27).

How D76N-β_2_m aggregates to form amyloid is less clear. The protein also folds *via* an I_T_ state that is structurally similar to that of WT-β_2_m and ΔN6-β_2_m (17), yet the rate of aggregation of D76N-β_2_m appears not to correlate with the concentration of I_T_ (17). Some have suggested an alternative native-like, but non-native species, N∗, might be responsible for the high aggregation potential of the protein (21, 28). There is also no information currently available about oligomers formed early in D76N-β_2_m amyloid formation, including how they relate to the on-pathway dimers described for ΔN6-β_2_m, or the inhibitory dimers formed between ΔN6-β_2_m and the nonaggregating murine β_2_m (mβ_2_m) (27, 29).

To investigate dimeric species formed from D76N-β_2_m, we here employ NMR and crosslinking mass spectrometry (MS) to identify, trap, and characterize dimers formed during D76N-β_2_m self-assembly. NMR is a versatile technique that allows exploration of the structure, dynamics, and the interactome of biomolecules in solution (30, 31). NMR in combination with paramagnetic relaxation enhancement (PRE) is ideal for identifying and structurally characterizing transient intermediates in biomolecular interactions with atomistic resolution, and herein, we use this approach to investigate transient intermolecular dimers formed for D76N-β_2_m (27, 32, 33). A number of paramagnetic probes are available to explore these transient states and *S*-(1-oxyl-2,2,5,5-tetramethyl-2,5-dihydro-1H-pyrrol-3-yl) methyl methanesulfonothioate (MTSL) is the most widely utilized in the field due to its relative ease of coupling to the target protein *via* native or introduced Cys residues (34). We further investigated the interacting interfaces of the transient dimers formed by applying a photo-crosslinker to trap the dimeric species (35, 36), allowing us also to investigate their role in aggregation. The results revealed an interaction interface for D76N-β_2_m dimers that is distinct from that observed previously for the on-pathway dimers of ΔN6-β_2_m. The crosslinked D76N-β_2_m dimers are not able to assemble into amyloid and are potent inhibitors of D76N-β_2_m assembly. The results highlight the significance of investigating short-lived intermolecular species in solution to better understand the roles they play in biological mechanisms (27). They also uncover an efficient strategy to inhibit amyloid assembly by targeting early dimers as kinetic traps of the assembly process.

## Results

### Exploring transient dimers of D76N-β_2_m in solution

To initiate our studies of D76N-β_2_m assembly, we measured the rate of aggregation of the protein into amyloid using thioflavin T (ThT) fluorescence and compared the behavior of the protein with that of WT-β_2_m. The experiments were performed at pH 6.2 in 25 mM sodium phosphate buffer supplemented with 115 mM NaCl—conditions identical to those used previously to determine the aggregation mechanism of ΔN6-β_2_m (27). Consistent with our previous results (16, 17), D76N-β_2_m aggregates into amyloid rapidly under these conditions, with a T_half_ (time to reach 50% of the maximum ThT signal) of 17.3 ± 2.4 h and reaching a plateau after ∼20 h, while WT-β_2_m did not form ThT-positive fibrils within the 42 h timescale of this experiment (Fig. 1*B*). Consistent with this, negative stain electron micrographs of the samples at the end of the experiment clearly displayed the presence of amyloid fibrils for D76N-β_2_m, while no detectable fibrils were observed for WT-β_2_m (Fig. 1*B* (inset)). Given the very similar native/I_T_ structures of the proteins (14, 17, 20, 26, 37), the dramatic differences in their amyloid potential suggests that early oligomers formed from the two proteins might differ significantly in conformation.

To explore the nature of transient noncovalent oligomeric species formed from D76N-β_2_m, 2D ^1^H-^15^N heteronuclear single quantum coherence (^HN^HSQC) NMR spectra were acquired at protein concentrations spanning 25 μM to 200 μM (in 25 mM sodium phosphate, 115 mM NaCl, pH 6.2, 25 °C). Surprisingly, and in marked contrast to the behavior of ΔN6-β_2_m (27), no significant changes in HN chemical shift or peak intensity were observed as a function of protein concentration over this range (Fig. 1*C*), indicating that dimeric or higher order oligomeric species, if formed, are not detected under these conditions.

**Figure 2:**
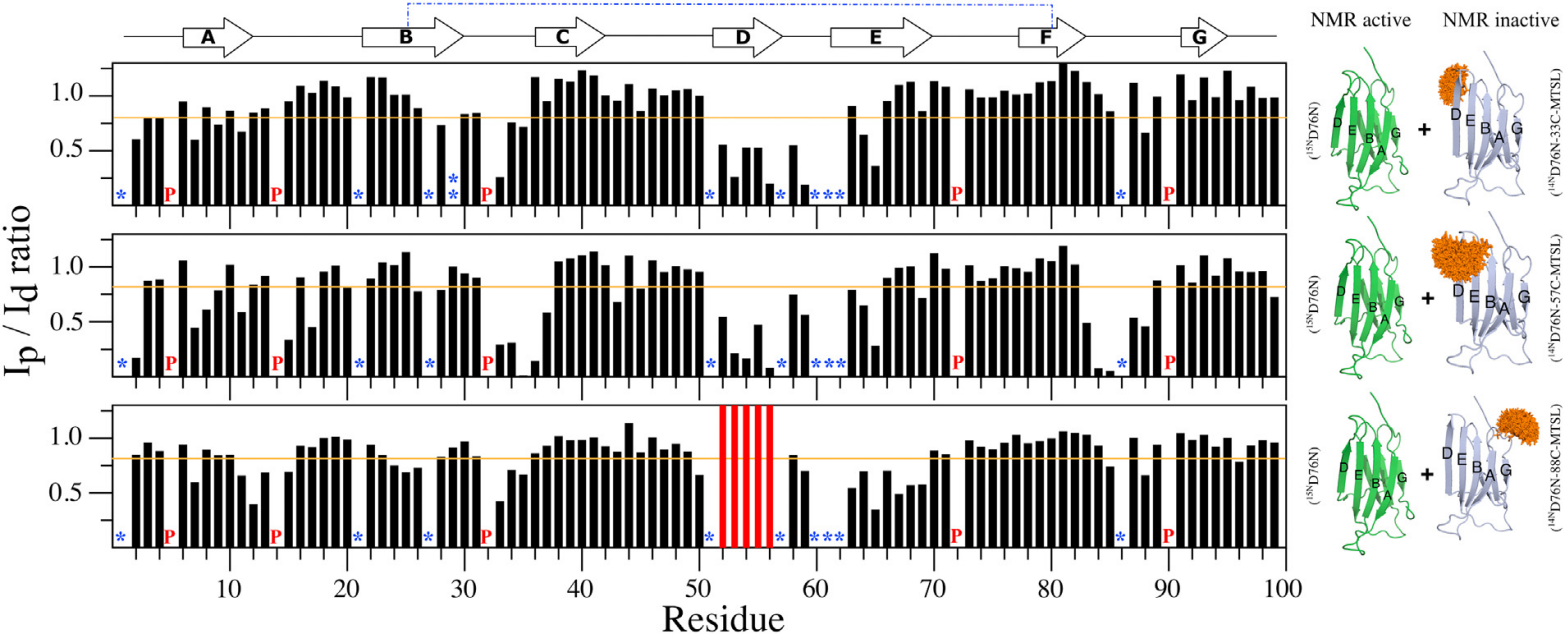
**Intermolecular HN-PREs of D76N-β2m at pH 6.2 and 25 °C.** 100 μM of ^15^N-D76N-β_2_m was mixed with 100 μM of each ^14^N-D76N-β_2_m-MTSL variant. The PRE is given as the amide crosspeak intensity ratio in 2D ^HN^HSQC spectra of ^15^N-D76N-β_2_m under paramagnetic (I_p_) *versus* diamagnetic (I_d_) conditions. Graphs from top to bottom refer to HN intermolecular PRE data of ^15^N-D76N-β_2_m in the presence of equimolar 33C, 57C, and 88C-MTSL labeled ^14N^D76N-β_2_m variants, respectively (see schematics alongside each graph, *green*^15^N-D76N-β_2_m, *gray*^14^N-D76N-β_2_m Cys variants, MTSL *orange stick*). *Vertical red bars* represent residues that had the strongest paramagnetic effect such that the resonance was not detectable in the oxidized spectrum. *Horizontal orange lines* depict the threshold value of 0.8. All values below the threshold were considered as significant. Missing data indicate residues that are (a) not assigned (I1, S57, W60, S61 and F62, T86), overlapped (Q21, V27, and H51), or proline (5, 14, 32, 72 and 90). The secondary structure of the native protein (β-strands from A to G) linked by the C25-C80 disulfide bond (*blue dotted line*) is shown at the top of the figure. Single and double asterisks refer to residues not assigned or HN-line broadened under diamagnetic conditions, respectively. P are prolines. Assignments have previously been reported (17) (BRMB accession code: 50302). β_2_m, β_2_-microglobulin, MTSL, S-(1-oxyl-2,2,5,5-tetramethyl-2,5-dihydro-1H-pyrrol-3-yl) methyl methanesulfonothioate; PRE, paramagnetic relaxation enhancement.

Given that oligomers of D76N-β_2_m were not detected in the experiments aforementioned, we next used NMR PREs to explore whether lowly populated, transient oligomeric states are formed that could play a role in governing the aggregation of D76N-β_2_m. NMR PREs are ideal to detect transiently formed intermolecular species, as they are able to detect species that populate between 0.2% to 0.5% of the total conformers in solution, especially when they are in dynamic equilibrium on a fast NMR timescale (38). Accordingly, MTSL was covalently coupled to a cysteine residue introduced into different locations on the surface of ^14N^D76N-β_2_m (NMR inactive, bait). The variants created were S20C (AB loop), S33C (BC loop), S57C (DE loop), and S88C (FG loop) (Fig. 1*A*). The new Cys residues were inserted into solvent-exposed loops in the protein, in order to avoid alterations in the structure of the native protein. Sequence changes in the major aggregation-prone region of the protein (residues 60–66), shown previously to be the only region to affect the rate of aggregation into amyloid using mutational scanning, were also avoided (16). Indeed, control experiments in which each Cys-containing protein was modified with (diamagnetic) MTSL showed that all variants are natively folded at the temperature of the NMR PRE experiments (25 °C), despite being thermodynamically destabilized (Figs. S1 and S2). Each MTSL-labeled protein at natural nitrogen abundance (^14^N) was then mixed 1:1 with ^15N^D76N-β_2_m (NMR active, target) and intermolecular PREs resulting from transient noncovalent interactions were measured for each variant (using 100 μM of each protein) (see schematics in Fig. 2). No significant intermolecular PREs were observed when ^14^N-D76N-β_2_m/S20C-MTSL was mixed with ^15N^D76N-β_2_m (Fig. S3), suggesting that if dimers form, they do not involve this region of the protein. By contrast, when ^14^N-D76N-β_2_m/S33C-MTSL, ^14^N-D76N-β_2_m/S57C-MTSL, or ^14^N-D76N-β_2_m/S88C-MTSL were each mixed individually with ^15N^D76N-β_2_m PREs were observed, with the strongest involving three regions in ^15N^D76N-β_2_m: a) residues ∼33 to 37 (the BC loop), b) residues 52 to 65 (D/E strands and DE loop) and c) residues 83 to 88 (FG loop) (Figs. 2 and S4). This suggests that one or more oligomer(s), probabilistically dimers, are transiently formed that involve interactions at these interfaces. In addition, residues 7 to 9 and 11 to 15 in the target protein also show a significant PRE effect (I_p_/I_d_ < 0.8), especially when the ^14^N-D76N-β_2_m/S57C-MTSL and ^14^N-D76N-β_2_m/S88C-MTSL probes were used (Fig. 2).

### Mapping the dimer interface using crosslinking

**Figure 3:**
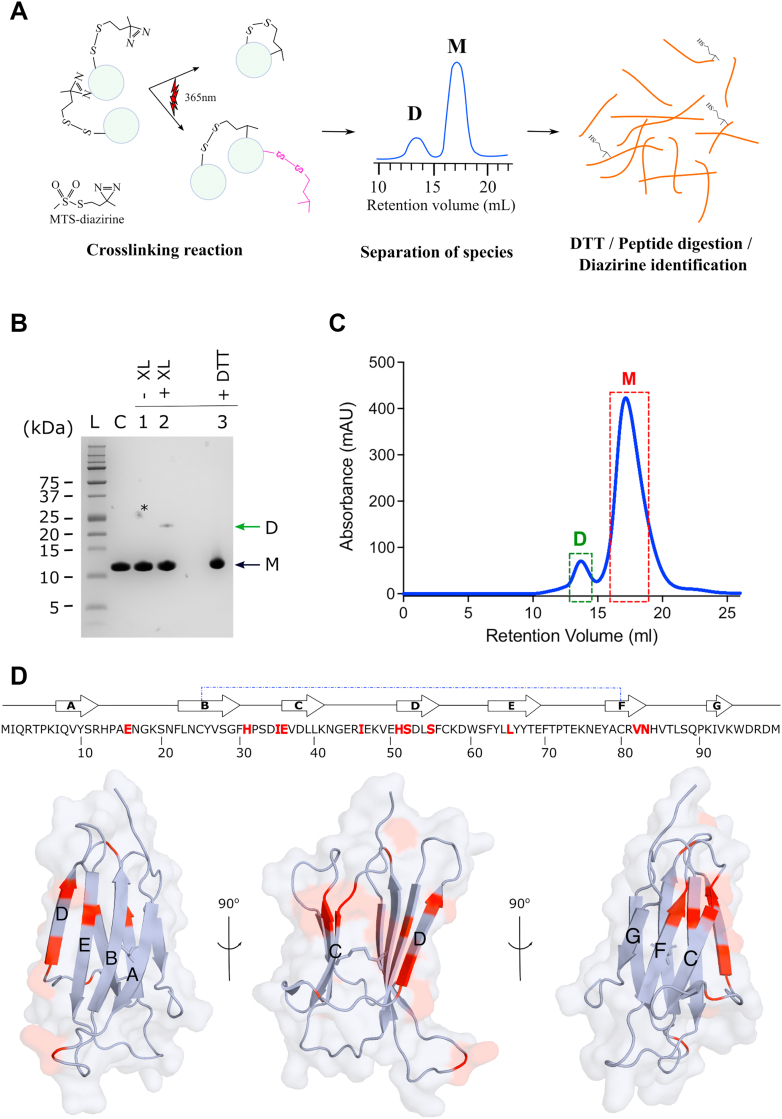
**Identification of D76N-β2m dimers *via* photo-crosslinking (XL).***A* schematic showing the structure of MTS-diazirine and the protocol used to identify crosslinked sites in dimers of D76N-β_2_m-S57C-diazirine. The reaction can induce the formation of crosslinked monomers and crosslinked dimers in which the addition diazirine has reacted intermolecularly/has not reacted/or has reacted with water (the latter shown here in *pink*). *B* analysis of the products of photo-crosslinking of D76N-β_2_m-S57C-diazirine using SDS-PAGE. Monomeric (M) and dimeric (D) species have an expected molecular weight of 12 kDa and 24 kDa, respectively. L protein ladder; C, monomeric D76N-β_2_m; -XL and +XL samples before and after crosslinking with UV illumination, respectively; +DTT, sample treated with 20 mM DTT; ∗, shows a gel background artefact. *C* isolation of the photo-crosslinked species (dimer (D) and monomer (M)) by gel filtration in 25 mM sodium phosphate and 115 mM NaCl, pH 6.2. The square boxes depict the fractions collected for analysis using LC-MS/MS. *D*, residues modified by tag transfer crosslinking are shown in *red* in the protein sequence and are mapped into the 3D structure of D76N-β_2_m below. Mass spectra of the peptides derived from D76N-β_2_m containing the crosslinking modifications are shown in Fig. S5 and the modified peptides identified are listed in Tables S1 and S2. β_2_m, β_2_-microglobulin.

To characterize the interface formed in the transient D76N-β_2_m dimers in more detail, we exploited a tag-transfer photo-crosslinking strategy, using a cleavable MTS-diazirine heterobifunctional crosslinker developed previously (Fig. 3*A*) (35, 36). This crosslinker is first attached to a specific Cys on the surface of one of the interacting partners in a protein–protein interaction, with the attached diazirine moiety allowing subsequent rapid and nonspecific crosslinks to heavy atoms, which lie within <10 Å of the Cα of the Cys modified with the crosslinker (36). Subsequent reduction and alkylation of the crosslinked species then enables identification of crosslinked sites *via* their covalent modification (addition of 145.06 Da) (Fig. 3*A*). Combined with the use of an LED illuminating system (36) high yields of crosslinks can be obtained within seconds, even for rapidly interconverting systems that exist in the microsececond-millisecond regime (36). D76N-β_2_m-S57C was chosen for this analysis since residue 57 is intimately involved in the dimer interface, according to the magnitude of NMR PREs observed from this residue (Fig. 2). Accordingly, D76N-β_2_m-S57C was modified with MTS-diazirine, and the modified protein (200 μM) was then crosslinked for 30 s before analysis of the products using SDS-PAGE (Fig. 3*B*). As predicted, proteins migrating with the mass expected of dimers (∼24 kDa) resulted from the crosslinking. Addition of DTT (20 mM) subsequent to crosslinking resulted in a lack of dimers and reappearance of monomers, consistent with successful tag transfer (Fig. 3*B*). The dimeric and monomeric crosslinked species were then purified using gel filtration (Fig. 3*C*), subjected to in-solution digestion with trypsin (after treatment with DTT), and identification of crosslinked products using LC-MS/MS. Comparison of the two samples allowed intramolecular *versus* intermolecular crosslinks to be identified. The results of this analysis (Figs. 3*D*, S5, Tables S1 and S2) yielded intermolecular crosslinks from residue 57 on one monomer to residues in the dimer interface located in the AB loop (E15), BC loop (His31, Ile35 and Glu36), and the D-, E-, and F-strands (His51, Ser52, Ser55, Tyr63, Leu64, Val82 and Asn83) in an adjacent protein. These sites are consistent with the intermolecular PRE-NMR results presented previously, suggesting that these regions of the protein form the epicenter of the dimer interface.

### Model of D76N-β_2_m dimers

**Figure 4:**
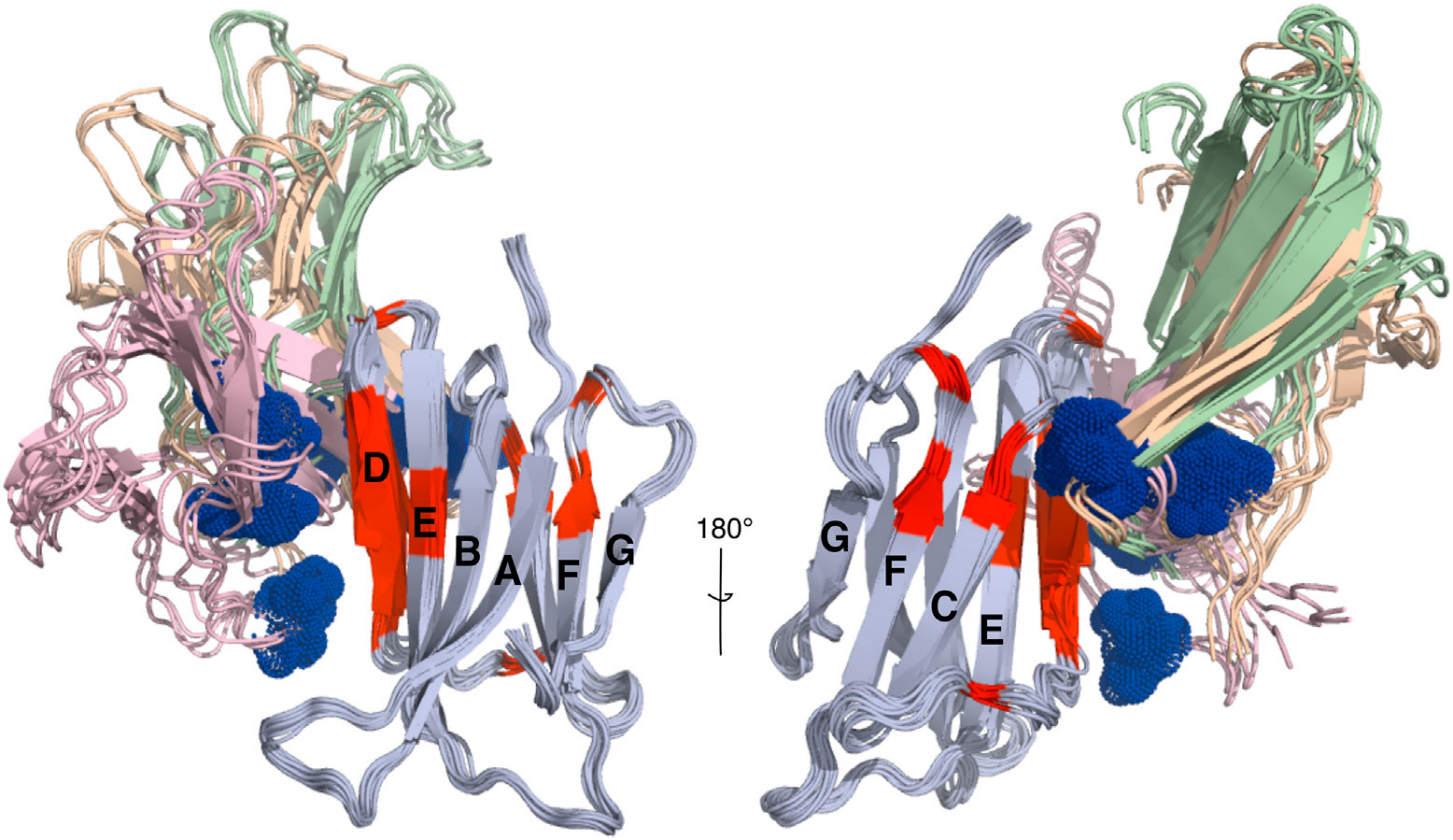
**Three-dimensional model of the dimers of D76N-β**_**2**_**m.** Different views of the three lowest energy clusters (1, 2, and 3)) of D76N-β_2_m dimers that best satisfy the NMR PRE and crosslinking restraints (Table S4). The bait monomer is oriented in three conformations (represented in *violet*, *light brown*, and *light green*) with respect to the target monomer (*light blue*). Restraints utilized to build the model are shown in *blue* (bait monomer) and *red* (target monomer). Each cluster has four representative structures. The dimer interface involves the BC-, DE-, and FG-loops from bait monomer and the D-strand and BC-loop from the target monomer. See also Fig. S6. Since the dimeric species of D76N-β_2_m is formed for three representative clusters of potentially many (related) structures, we cannot conclude that one dimer cluster is better than another. Clusters 1, 2, and 3 are shown here (in *violet*, *light brown*, and *light green*, respectively). β_2_m, β_2_-microglobulin.

In order to extract structural information on the transient dimers of D76N-β_2_m, rigid-body *in silico* docking was performed in explicit solvent using the NMR PRE and crosslinking data from S33C, S57C, and S88C as constraints (Table S3). All constraints were treated as nonambiguous. Data from S20C were not used for deriving the structural model, as no significant NMR PREs resulted from this site. The results revealed that three clusters (formed of four representative structures) satisfied the experimental constraints (Tables S4 and S5). In all of these clusters, the monomers involved in the model dimers adopt a ‘top-to-side’ orientation with respect to each other. The dimerization interface is dominated by residues in the BC loop (Ile35 and Glu36), D-strand (His51, Ser52, Asp53, Leu54, Ser55, Phe56 and Asp59), and E-strand (Leu54 and Leu55) from one monomer and BC (Ser33), DE (Ser57), and FG (Ser88) loops from the other monomer (Fig. 4). These dimers differ significantly from the on-pathway ‘head to head’ homodimers of ΔN6-β_2_m (27) and ΔN6-β_2_m:mβ_2_m heterodimers (29) (Fig. S6), as well as the canonical ‘edge strand-edge strand’ organization of Ig domain dimers in IgGs (39). Notably, the distance of Cys20 to the dimer interface in the models generated is >30 Å and hence is outside the sensitivity of the PRE, consistent with a lack of significant PREs from this site (Fig. S3).

### D76N-β_2_m crosslinked dimers are effective inhibitors of amyloid formation

**Figure 5:**
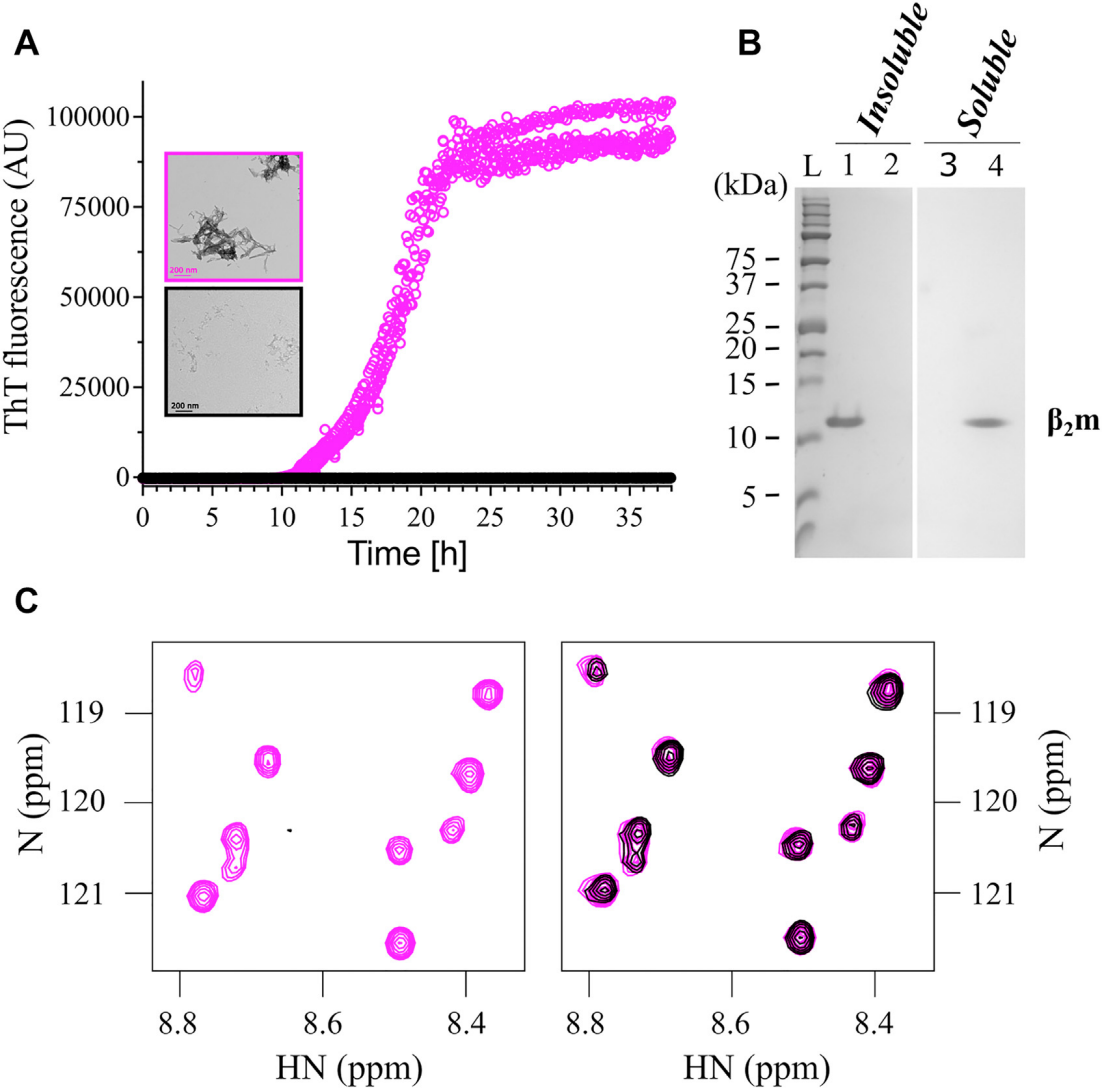
**Efficient inhibition of fibril formation of D76N-β**_**2**_**m by D76N/57C-diazirine-β**_**2**_**m crosslinked dimers.***A* ThT fibrillation kinetics of ^15^N-D76N-β_2_m (20 μM) with (*black*) or without (*pink*) 2 μM (dimer equivalent concentration) of crosslinked dimers (^14^N). Negative stain electron micrographs of each sample after 38 h of fibrillation are shown inset (scale bar = 200 nm). Each experiment was performed in three replicates. *B* SDS-PAGE analysis of soluble and insoluble fractions at the end of the fibrillation reaction. Insoluble fractions of ^15^N-D76N-β_2_m without (lane 1) or with 10 % (*w/w*) crosslinked dimer (lane 2), with the corresponding soluble fractions in lanes 3 and 4. M is the protein ladder (kDa). *C*, example region of 2D ^HN^HSQC-NMR spectra of ^15^N-D76N-β_2_m (20 μM) at the start of the incubation (*pink*) and after 38 h (*black*) incubation in a plate reader either without (*left*) or with (*right*) crosslinked dimer. The results show that the presence of dimer does not alter the spectrum (chemical shift or linewidth) suggestive of a weak interaction that is able to inhibit assembly, yielding native monomeric protein at the end of the reaction. By contrast no visible protein is detectable in the absence of crosslinked dimers at the end of the incubation time, since the protein has formed amyloid fibrils. All spectra were acquired at 25 °C on a 950 MHz spectrometer. Full spectra are shown in Fig. S9. β_2_m, β_2_-microglobulin; ThT, thioflavin T.

Previous studies have shown that dimers of WT-β_2_m crosslinked by disulfide bonds at residue 33 (Fig. S6) accelerate amyloid formation of the normally aggregation-resilient protein (at least in the presence of the cosolvent trifluoroethanol) (40). We therefore analyzed how the purified crosslinked dimers (XL-Ds) of D76N-β_2_m/S57C affect amyloid formation, by incubating the dimers either alone or in the presence of monomeric D76N-β_2_m and monitoring amyloid formation *versus* time *via* ThT fluorescence (20 μM monomeric protein in 25 mM sodium phosphate, 115 mM NaCl, pH 6.2). Under these conditions, D76N-β_2_m forms fibrils rapidly (T_half_ of *ca.* 17.3 ± 2.4 h, with the maximal ThT signal after ∼20 h) (Fig. 5). Surprisingly, fibril formation was arrested over the total incubation time when 10% (*w/w*) crosslinked dimer was added (Fig. 5*A*), with significant retardation in the kinetics of fibril growth (0.8-fold increase in T_half_) occurring when as little as 1.25% (*w/w*) dimer was added (Fig. S7, *B* and *D*). Notably, the XL-Ds alone were unable to assemble into ThT-positive amyloid (Fig. S7, *B* and *D*) but did aggregate into material able to be pelleted by centrifugation (Fig. S8) when incubated alone at high concentration, presumably since the conformational changes required to form a cross-β structure are not possible from the crosslinked state. In marked contrast with the behavior of the XL-Ds on fibril formation, addition of the crosslinked monomers marginally increased the rate of fibril growth, demonstrating that the inhibition is specific to the dimeric species (Fig. S7, *A* and *C*).

To confirm the inhibition of fibril growth by the XL-Ds, insoluble and soluble material after 120 h of incubation of each sample was separated by centrifugation and the percent insoluble material quantitatively determined by SDS-PAGE (Experimental procedures). The results showed that D76N-β_2_m incubated without XL-Ds was found exclusively in the insoluble fraction after 120 h of incubation (Fig. S8, *A* and *B*). D76N-β_2_m incubated with the intramolecularly crosslinked monomers also showed no decrease in aggregate yield. However, in samples that contained >5 % (*w/w*) XL-Ds, little, if any, insoluble material could be detected (Figs. 5*B*, S8, *A*, and B), demonstrating the unique ability of the XL-Ds to arrest amyloid assembly.

### Defining the inhibition mechanism of D76N/57C-β_2_m dimers

To examine in more detail the mechanism by which XL-Ds are able to arrest amyloid assembly of D76N-β_2_m, the soluble products of the inhibition reaction were examined using ^HN^HSQC NMR. For these experiments, monomeric ^15^N-labeled D76N-β_2_m was mixed with 10 % (*w/w*) ^14^N-labeled XL-Ds and a ^HN^HSQC NMR spectrum immediately recorded. The sample was then removed from the NMR tube, placed in a 96-well plate, and incubated under identical conditions to those used for the ThT kinetic analysis. After 38 h, the sample was removed and a ^HN^HSQC NMR spectrum again recorded. The results revealed no effect on the chemical shifts and only a minor increase in linewidth (∼12% reduction across all resonances) of the monomeric ^15^N-labeled protein when the samples were immediately mixed, consistent with a weak interaction between the ^15^N-monomers and XL-Ds (Fig. S9). The chemical shifts and linewidths of the ^15^N-labeled protein product at the end of the incubation were also unchanged relative to the starting material (Fig. 5*C*). These results demonstrate that the XL-Ds kinetically inhibit aggregation by transient binding to monomers, rather than by stabilizing unproductive larger oligomeric species, as has been observed with small molecule inhibitors of β_2_m amyloid formation (41, 42).

**Figure 6:**
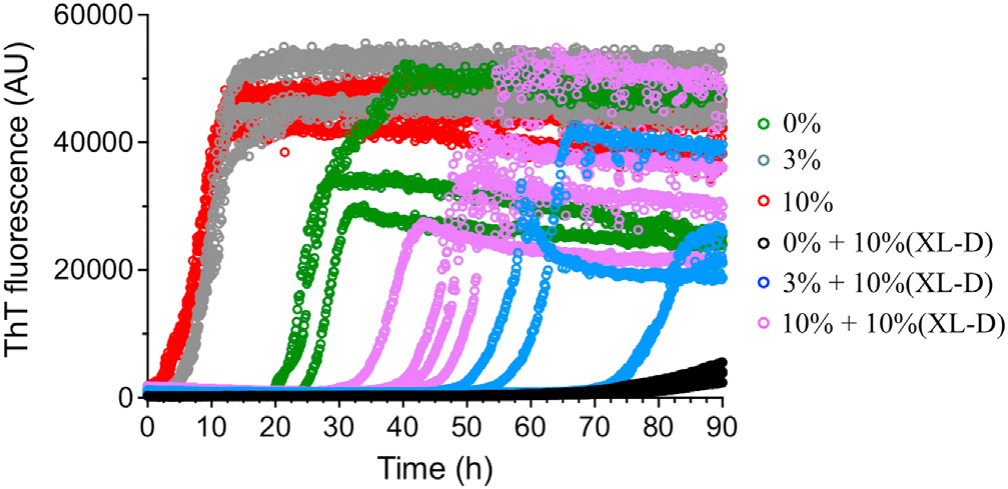
**D76N-β**_**2**_**m fibrillation assay in presence of different percentages of preformed D76N-fibril seeds and crosslinked dimers.** Fibrillation kinetics of monomeric D76N-β_2_m (20 μM) in the absence or presence of 0%, 3%, or 10% (*w/w*) preformed fibril seeds (*green*, *gray*, and *red*, respectively). To equivalent samples, 10 % (*w/w*) of purified crosslinked dimers (XL-Ds) were added and the kinetics of amyloid formation monitored using ThT fluorescence (*black*, *blue* and *violet* traces, respectively). The experiments show the ability of the crosslinked dimers to inhibit seeded assembly. All experiments were performed at 37 °C with shaking (600 rpm) in 25 mM sodium phosphate, 115 mM NaCl, pH 6.2. *D*, crosslinked dimer. Each experiment was performed in four replicates. β_2_m, β_2_-microglobulin; ThT, thioflavin T.

To explore whether the XL-Ds are also able to kinetically inhibit fibril elongation, fibril growth was measured in the presence of seeds formed from preformed D76N-β_2_m fibrils (Experimental procedures). The T_half_ of fibril formation is decreased (∼2.5- and 2.7-fold) in the presence of 3% or 10% (*w/w*) fibril seeds, respectively, consistent with elongation and/or secondary nucleation mechanisms enhancing the rate of fibril formation (Fig. 6). When supplemented with 10% (*w/w*) XL-Ds, fibril growth in the presence of seeds was again inhibited (relative to the same reactions in the absence of XL-Ds), with the magnitude of inhibition depending on amount of seeds added (0.8- and 1.6-fold increase in T_half_ in the presence of 3 % and 10% (*w/w*) fibril seeds and 10% (*w/w*) crosslinked dimer, respectively, relative to the unseeded reactions plus dimer). Clearly, therefore, XL-Ds are able to retard seeded fibril growth, consistent with binding to monomers and/or the fibrils themselves.

To determine whether the XL-Ds bind to preformed fibrils, a pelleting assay was performed in which preformed fibril seeds were incubated with XL-Ds and binding measured by centrifugation to separate fibril bound *versus* free XL-Ds (Experimental Procedures). The results (Fig. S10*A*) revealed that the XL-Ds do not interact stably with fibrils. Finally, surface plasmon resonance experiments, in which D76N-β_2_m monomers were immobilized onto the chip and XL-Ds passed over the surface, were used to confirm that the XL-Ds do indeed bind to monomeric D76N-β_2_m (Experimental procedures) (Fig. S10*B*).

Together with the data presented previously, the results indicate that the XL-Ds are able to inhibit primary nucleation— similar behavior to the action of some molecular chaperones (43). Additionally, elongation and/or secondary nucleation processes involved in the fibrillation of D76N-β_2_m are also inhibited by XL-Ds. Such kinetic inhibition presumably results from transient binding of the XL-Ds to D76N-β_2_m monomers, reducing the effective concentration of assembly competent monomers for participation in fibril growth events. Such a mechanism would also explain the effectiveness of the XL-Ds to inhibit assembly when added in substoichiometric amounts. However, other mechanisms of action, including interaction with oligomeric species not visible in the experiments used here, cannot be ruled out.

## Discussion

Protein self-assembly is a ubiquitous, and often complex, process involved in an array of functions in biology (44, 45). While assembly reactions can create protein complexes that endow new function(s), uncontrolled or aberrant self-assembly can lead to disastrous consequences for the cell (46). Amyloid formation is one example of such aberrant self-assembly, which results in the formation of highly ordered cross-β fibrils associated with hundreds of human diseases (46, 47) (although amyloid can also have functional roles (46, 48, 49)). While reversing amyloid assembly by depolymerizing assembled fibrils is challenging, but possible (50, 51), because of the tremendous thermodynamic stability of the amyloid fold (52, 53), slowing amyloid formation by kinetically inhibiting the assembly steps of nucleation, fibril elongation, and/or secondary nucleation are more tractable. Strategies to inhibit fibril assembly using small molecules (54, 55, 56, 57), chaperones (58, 59), nanobodies (60, 61) and other protein scaffolds (62, 63, 64) have been reported, with several studies demonstrating their efficacy *in vitro*, in cells and/or in model organisms (61, 65). Indeed, kinetic stabilization of the native tetramer of transthyretin has resulted in the first small molecule therapy against a human amyloid disease (66).

Here, we describe the ability of stabilized dimers of an aggregating protein to inhibit its own self-assembly. We show that the mechanism of action of the inhibitory dimers is by transient binding to assembly competent monomers, which competes with the protein–protein interactions required for amyloid assembly. While populated only transiently and rarely in solution, the formation of D76N-β_2_m dimers presumably contributes to the observed rate of amyloid formation, with XL-Ds slowing assembly by binding to monomers and also by disfavoring the structural transitions needed to generate the amyloid fold. Analysis of the assembly mechanism of ΔN6-β_2_m into amyloid using NMR PREs, combined with detailed kinetic analysis using ThT fluorescence and other methods, identified head-to-head elongated dimers as essential precursors of amyloid formation, with the rate of amyloid formation dependent on the formation of these key initiating species (27). Interestingly, the on-pathway ΔN6-β_2_m dimers contain some interacting residues in common with those of the D76N-β_2_m inhibitory dimers identified here (namely the BC loop and partially the DE loop) but are structurally distinct in that the D76N-β_2_m dimers are asymmetric and involve mainly the D-strand in one monomer and BC-, DE-, and FG-loops on the other monomer (Figs. 4 and S6). These structural differences presumably define the diametrically opposed outcomes of assembly of these two dimer folds. Inhibitory dimers formed between ΔN6-β_2_m and mβ_2_m have also been reported previously (29). These heterodimers also involve head-to-head interactions of the BC and DE loops, with amyloid assembly also being inhibited when mβ_2_m is added in substoichiometric amounts (29). Inhibition of amyloid formation of ΔN6-β_2_m has also been achieved by covalent attachment of a small molecule fragment to residue 52 in the D strand, which results in the formation of off-pathway tetramers (41). These species were able to be crystallized, with the resulting structure providing a rationale for why this conformation is incompatible with generation of amyloid (41). Given this information, the inhibitory potential of the D76N-β_2_m dimers determined here can also be rationalized, at least in part, since they sequester interactions known to be essential for amyloid formation in their ΔN6-β_2_m counterparts. While further work will be needed to determine the structure of on-pathway dimers and higher order oligomers of D76N-β_2_m, the finding that regions involved in early assembly are shared in D76N-β_2_m and ΔN6-β_2_m suggests that the two proteins assemble *via* similar pathways, despite their involvement in distinct diseases that affect different individuals (with/without renal dysfunction), and result in amyloid deposition in different regions of the body (14, 67).

A second striking finding of the results presented here is the efficient nature of the inhibition caused by D76N-β_2_m XL-Ds, with complete arrest of assembly over 120 h being achieved with ratios of D76N-β_2_m:dimers of only 1:0.1 (*w/w*). We showed that inhibition occurs *via* kinetic competition, with dimer–-monomer interactions competing with productive monomer–monomer and monomer–fibril interactions that define primary nucleation, secondary nucleation, and fibril elongation. While the affinity of these different interactions for D76N-β_2_m remain unknown, previous studies of ΔN6-β_2_m:mβ_2_m heterodimers and ΔN6-β_2_m homodimers show K_d_s ranging from 68 to 494 μM (29), similar in magnitude with the weak intermolecular D76N-β_2_m interaction observed here. These results highlight the ability of weak binding to effect inhibition in these kinetically controlled assembly processes. Discovery of new amyloid inhibitors, therefore, will not necessarily require tight binding, as is the general case in ligand discovery strategies in medicinal chemistry (68). Instead, as we portray here, mapping the interacting interfaces in early oligomers, using NMR PREs and chemical crosslinking can provide an excellent starting point to generate strategies to disrupt amyloid fibril formation.

By contrast with the inhibition of amyloid formation shown here for D76N-β_2_m XL-Ds, crosslinking β_2_m or other proteins with disulfide bonds has been shown to accelerate amyloid formation, dependent on the location of the disulfide bond introduced (39, 65). This highlights the specificity of the interactions that kinetically define amyloid formation, at least for the β_2_m family of proteins (27, 69, 70). By contrast with the highly defined crosslinks involving disulfide bond formation, crosslinking *via* diazirines provides the opportunity of mapping more promiscuous or transient interactions. As we showed previously, it is also important to ensure that the covalent addition of new chemical moieties onto a protein sequence does not affect its structure or aggregation, as changes in solubility, local or global stability, and the inherent amyloid propensity of the sequence can all result from posttranslational or other chemical modifications. We demonstrate the structural integrity of labeled proteins herein using CD, NMR, and thermal stability measurements. Importantly, previous studies of 56 variants of D76N-β_2_m, obtained using random mutagenesis and screening for sequences with altered aggregation potential, have revealed that the presence of a single region, spanning residues 60 to 66, alongside Asn at residue 76, is essential for amyloid formation, with mutations elsewhere in the protein having little effect (16). Importantly, the inhibitory dimers identified here do not involve this critical region (which forms the E-strand in the native structure (Fig. 1*A*)).

Finally, our work has important implications for the amyloid field, by highlighting the potential of stabilizing early assembly intermediates as routes to inhibit amyloid formation. Such findings may provide new avenues to combat disease, by stabilizing lowly populated species of the same system and reusing their stable equivalents as possible therapeutic reagents. At a more fundamental level, the results highlight the specificity of the early interactions that drive β_2_m amyloid assembly, with different dimers able to promote or prevent amyloid formation in this family of proteins, dependent on the interactions made and their stability.

## Experimental procedures

### Protein production

The gene encoding WT-β_2_m and D76N-β_2_m are inserted in pINK expression vector (71). Protein expression was performed in BL21-(DE3) *Escherichia coli* and protein was purified as previously described (27). Monomeric protein was purified using size-exclusion chromatography, and the purity and fidelity of the sequence were determined using 12% SDS-PAGE and electrospray ionization MS (ESI-MS), respectively. ESI-MS also confirmed the formation of the disulfide bond in all samples. Monomeric proteins were divided into aliquots and frozen using liquid nitrogen and stored at −20 °C.

The S20C, S33C, S57C, and S88C variants of D76N-β_2_m were produced by site-directed mutagenesis and purified as aforementioned, except that an additional anion-exchange (Q-Sepharose) chromatography step under denaturing conditions (25 mM Tris–HCl, 8 M urea, pH 8.0) was included at the beginning of the purification. All Cys variants were refolded by flash dilution (1:10) in 25 mM Tris–HCl, 300 mM NaCl, 500 mM arginine, pH 8.0. The sample was then dialyzed three times in 25 mM Tris–HCl, pH 8.0.

### Fibrillation assays

D76N-β_2_m at natural nitrogen abundance (^14^N-D76N-β_2_m) or labeled with ^15^N (^15^N-D76N-β_2_m) were thawed at room temperature (RT) and then further purified by gel filtration. Monomeric fractions from size-exclusion chromatography (Superdex75 PG-26/600) were collected and immediately used for fibrillation assays under the following conditions: 20 μM of D76N-β_2_m (in the absence or presence of various concentrations of ^14^N-D76N-β_2_m-S57C–crosslinked dimer or monomer), 10 μM of ThT in 25 mM sodium phosphate, 115 mM NaCl, pH 6.2, in a 96-well plate (clear flat bottom, Corning 3631). The plates were sealed with a plastic film, incubated at 37 °C for 38, 90, or 120 h, depending on the conditions to be evaluated, with shaking at 600 rpm (Fluostar Omega plate reader). Amyloid formation was monitored by ThT fluorescence (excitation at 440 nm and emission at 475 nm). Fibril seeds were created by treating the samples at the end of a fibrillation reaction for 1 min of bath sonication at fixed frequency (Ultrawave instrument, model U100H).

Aggregate yield was determine using Tris-Tricine-SDS PAGE (5:1 ratio of acrylamide and bis-acrylamide, respectively) following separation of insoluble/soluble material by centrifugation (23,000*g* for 10 min). Pellets were treated with 8 M urea for 30 min in the same buffer before analysis by SDS-PAGE. In the case of the soluble material, no urea was added prior to SDS-PAGE. Gels were stained with instant blue solution (Abcam: ab119211), imaged, and band quantified using densitometry with UVITEC transilluminator (Q9Aliance).

### Electron microscopy

After the fibrillation, samples were collected and stored at 4 °C for analysis by electron microscopy. Ten microliters of fibril solution was added to a carbon-coated grid (home produced, without glow discharge) for 2 min, followed by staining with 2% (*w/v*) uranyl acetate. The grids were washed twice with water; electron micrographs were acquired on T12 microscope (Gatan US4000 equipped with 4k CCD camera and 120 keV Lab6 electron source) at the Electron Microscopy facility in the Astbury Biostructure Laboratory University of Leeds.

### ^HN^HSQC NMR and data processing

^HN^HSQC spectra, obtained using eight scans, 2048 and 164 complex points in the direct and indirect dimensions, respectively, were acquired on Bruker 750 MHz or 950 MHz NMR spectrometers, equipped with TCI (^1^H, ^2^H, ^13^C, ^15^N channels) cryoprobes (Bruker Avance III HD console, acquisition software, Topspin 3.2). All spectra were recorded at 25 °C, pH 6.2, in 25 mM sodium phosphate, 115 mM NaCl supplemented with 3% (*v/v*) D_2_O using protein concentrations ranging from 20 to 200 μM. NMRPipe (72, 73) was used to process and analyze the spectra and peak intensity was extracted using *Analysis2.4.2*-ccpNMR software (https://ccpn.ac.uk/software/version-2/) (74). NMR assignments were taken from BMRB (access code: 50302), which were obtained under identical conditions to those employed here.

### MTSL labeling

^14^N-D76N-β_2_m Cys variants (S20C, S33C, S57C, and S88C) were thawed at RT. The samples were incubated with 20 mM DTT for 30 min at RT. DTT was then removed from solution using a 5 ml centrifugal desalting column (Zebra 7K/MWCO, 89882), which was equilibrated with 50 mM sodium phosphate and 150 mM sodium chloride, pH 7, prior to use. Eluted protein fractions were quantified and the protein concentration was adjusted to 200 μM. The sample was then mixed with 2 mM (final concentration) of MTSL (CAS: SC-208677) in 500 mM guanidine HCl. The reaction mixture was incubated for 4 h at RT and then centrifuged at 44,000*g* for 1 h to remove any protein aggregates. β_2_m-Cys-MTSL labeled variants were isolated by gel filtration (Superdex75 PG-26/600), and monomeric fractions were collected and stored at 4 °C for further use. The mass of the modified proteins was confirmed by ESI-MS recorded using a Xevo QToF G2-XS mass spectrometer (Waters UK) operated in positive ion mode. The MS spectra show complete labeling of the proteins (observed masses of D76N-β_2_m of 12,059.46 ± 0.28 Da, 12,059.78 ± 0.11 Da for S57C-MTSL and S88C-MTSL, respectively (expected mass of 12060 Da).

### Thermal denaturation

Changes in the stability of D76N-β_2_m as result of insertion of cysteine and MTSL at residues 20, 33, 57, and 88 were evaluated by thermal denaturation using far UV CD (Chirascan Plus). For these experiments, 20 μM of protein, dissolved in 25 mM sodium phosphate, 115 mM NaCl, pH 6.2 was used. The temperature ramp was from 20 °C to 80 °C, at a rate of 1 °C/min. A far UV CD spectrum (195–260 nm) of each protein was acquired at each temperature during the ramp. The data were fitted to two state equilibrium in CDPal software (https://github.com/PINT-NMR/CDpal) (75).

$$E=e^{-\left(\frac{\Delta Hm}{R}\right)\left(\frac{1}{Tm}-\frac{1}{T}\right)-\left(\frac{\Delta Cp}{R}\right)\left(\frac{Tm}{T}-1+\ln\left(\frac{T}{Tm}\right)\right)}$$

Where *E* is the mean residue ellipticity, *ΔH*_*m*_ is the change of enthalpy at the midpoint of denaturation (*Tm*), *ΔCp* is the change of heat capacity, *R* the universal gas constant, and *T* is the temperature (Kelvin).

## NMR PRE experiments

To measure intermolecular PREs, 100 μM ^15^N-D76N-β_2_m was mixed with 100 μM of ^14^N-D76N-S/C-MTSL variants and ^HN^HSQC spectra were recorded (paramagnetic conditions) at 25 °C in 25 mM sodium phosphate, 115 mM NaCl, pH 6.2. Ascorbic acid dissolved in the same buffer at pH 6.2 was then added to a final concentration of 1 mM and left at RT for 1 h. A second ^HN^HSQC spectrum was then collected (diamagnetic conditions). The NMR data were processed as described previously. Note that no significant change in pH is observed after addition of ascorbic acid (<0.1 units) indicated by measuring the pH of the solution and analysis of the chemical shifts (HN) of His residues. Analysis of chemical shift perturbations of the diamagnetic samples were determined using the equation:

CSP=(5ΔδH1)2+(ΔδN15)2

These experiments showed no significant change in structure of each monomer, compared with unmodified D76N-β_2_m (Fig. S2). The extent of the intermolecular PRE effect was evaluated by calculating the ratio (I_p_/I_d_) of resonance intensity of the paramagnetic (I_p_) and diamagnetic (I_d_) samples. The conformational ensembles of MTSL shown in different figures were generated using the MTSLWizard module in PyMol (76).

### Tag transfer photo-crosslinking

^14^N-D76N-β_2_m-S57C (200 μM protein) was labeled with the MTS-diazirine photo-crosslinker (Fig. 3*A*) using a protocol similar to that of MTSL labeling. The reaction was left to progress for 4 h at RT. Then, MTS-diazirine–labeled monomeric protein was recovered by gel filtration. The mass of the modified proteins was confirmed by ESI-MS (11,905 ± 0.37 Da, expected mass for the labeled monomer 11,905 Da).

Photo-crosslinking was achieved at RT using 200 μM ^14^N-D76N-β_2_m-S57C-diazirine in 25 mM sodium phosphate and 115 mM NaCl, pH 6.2. The sample was photo-crosslinked for 30 s at 365 nm using a home-built LED illumination device (36). Crosslinked species were isolated by gel filtration (Superdex75 Increase 10/300) equilibrated with 25 mM sodium phosphate and 115 mM NaCl, pH 6.2. Monomeric and dimeric crosslinked conformers were verified by 12% Tris-Tricine-SDS-PAGE (5:1 ratio, acrylamide and bis-acrylamide, respectively). Finally, samples were stored at 4 °C.

## Identification of crosslinked sites using trypsin digestion and MS

The isolated pure crosslinked dimer and monomer samples were first reduced by incubation with 10 mM DTT at 57 °C for 1 h with shaking. The samples were left to cool to RT, followed by alkylation with 55 mM iodoacetic acid at RT for 45 min in the dark with shaking. Then, trypsin (20 ng/μl in 25 mM ammonium bicarbonate) was added in a 1:50 ratio (*w/w* of protease:β_2_m) and the mixture was incubated at 37 °C for 18 h with shaking. The reaction was stopped by adding 5 μl of 1% (*v/v*) TFA. The digested peptides were purified by reverse phase chromatography on Sep-Pak C18 column. Peptides were eluted from the column with 500 μl 50% (*v/v*) acetonitrile and 0.1% (*v/v*) formic acid. Finally, the peptides were evaporated to dryness and reconstituted in 20 μl 0.1% (*v/v*) aqueous TFA prior to MS analysis.

The peptide-containing solution (3 μl) was injected into a reverse-phase in house-packed C18) capillary column (75 μm × 200 mm) and separated by gradient elution of 5% to 95% (*v/v*) acetonitrile with 0.1% (*v/v*) formic acid at a flow rate of 250 nl/min. The separated peptides were eluted directly from the column and then infused into an Orbitrap Velos (ThermoFisher Scientific) mass spectrometer using an electrospray capillary voltage of 2.7 kV. The mass spectrometer was operated in positive ion mode. Data acquisition was performed in data-dependent acqusition mode and fragmentation was performed by using ion-trap. Up to 20 most intense ions per precursor scan were selected for MS/MS. Dynamic exclusion of 30 s was used. Peptide MS/MS data processing and modification localization were performed by using PEAKS Studio X^+^ (Bioinformatic Solutions Inc).

### Modeling dimers of D76N-β_2_m using docking

Two molecules of D76N-β_2_m (Protein Data Bank: 4FXL) (14) were used as input for flexible molecular docking. One molecule was modified by the insertion of three cysteines at positions 33, 57, and 88. PRE (HN) and crosslinking data were then used as experimental restraints: a radius of 7.5 Å (covering the distance between the sulfhydryl group and the nitroxy group in MTSL) or 12 Å (covering the distance between sulfhydryl [bait molecule] and nitrogen [target molecule] atoms for crosslinking) was employed. The second molecule was not modified. PRE restraints were organized into three groups according their I_p_/I_d_ ratio value (I_p_/I_d_ 0.2–0.6, 0.05–0.2 and < 0.05 [resonance undetectable]) (Table S3). 4FXL-Cys (modified monomer, A) and 4FXL (unmodified monomer, B) were used as input structures for molecular docking using HADDOCK2.4, allowing flexibility in all segments (77). The docking process was performed in three sequential steps: rigid body, semiflexible, and finally, water refinement. Two-hundred structures were analyzed and then clustered in three families (with four representative members). Sampling and clustering parameters during docking are summarized in Table S4, and the number of restraints satisfied/not satisfied by the final lowest energy structures are shown in Table S5.

### Binding of XL-Ds to fibril seeds

D76N-β_2_m fibrils were freshly generated in 25 mM sodium phosphate and 115 mM NaCl, pH 6.2, as described previously. Fibrils were then recovered by centrifugation at 13,000 rpm (benchtop microfuge) and their concentration adjusted to 20 μM (monomer equivalent concentration) in the same buffer. XL-Ds (2 μM) were added and incubated for 1 h at RT by centrifugation (13,000 rpm, 10 min), and the XL-D remaining in solution were quantified by measuring the absorbance at 280 nm.

### Binding of XL-D to monomers

Surface plasmon resonance was used to assay monomer:XL-D interactions. For this assay, a cysteine was inserted into D76N-β_2_m immediately after the initiating N-terminal methionine (Cys0). The sample was then labeled with EZ-Link-Maleimide-PEG11-Biotin (ThermoFisher: 21911) using a protocol similar to that used for labeling with MTSL or diazirine, as described previously. Binding of XL-D to monomers was monitored using a Biacore T200 instrument (Cytiva). D76N-C0-Biotin-β_2_m was immobilized onto a streptavidin sensor chip (Cytiva-BR100398) and XL-D was passed over the immobilized monomer. All samples were in 25 mM sodium phosphate and 115 mM NaCl, pH 6.2. Nine concentrations of XL-D were tested between 5.8 nM and 1.5 μM (Fig. S10*B*). The association of XL-D was monitored for 60 s, followed by 360 s of dissociation at a flow rate of 30 μl/min at 25 °C.

## Data availability

Data are available upon requested and are deposited in the DOI: https://doi.org/10.5518/1159.

## Supporting information

This article contains supporting information (14, 26, 40).

## Conflict of interest

The authors declare there they have conflict of interest with the contents of this article.

## References

[1] Saper M.A., Bjorkman P.J., Wiley D.C. (1991). Refined structure of the human histocompatibility antigen HLA-A2 at 2.6 Å resolution. J. Mol. Biol..

[2] Bjorkman P.J., Parham P. (1990). Structure, function, and diversity of class I major histocompatibility complex molecules. Annu. Rev. Biochem..

[3] Otten G.R., Bikoff E., Kozlowski S., Margulies D.H., Germain R.N., Germain R.N. (1992). Peptide and beta 2-microglobulin regulation of cell surface MHC class I conformation and expression. J. Immunol..

[4] Cresswell P., Springer T., Strominger J.L., Turner M.J., Grey H.M., Kubo R.T. (1974). Immunological identity of the small subunit of HLA antigens and β_2_ microglobulin and its turnover on the cell membrane. Proc. Natl. Acad. Sci. U. S. A..

[5] Gejyo F., Odani S., Yamada T., Honma N., Saito H., Suzuki Y. (1986). β_2_-microglobulin: a new form of amyloid protein associated with chronic hemodialysis. Kidney Int..

[6] Gejyo S.T., Yamada T., Odani S., Nakagawa Y., Arakawa M., Kunitomo T. (1985). A new form of amyloid protein associated with chronic haemodialysis was identified as β_2_-microglobulin. Biochem. Biophys. Res. Commun..

[7] Gorevic P.D., Munoz P.C., Casey T.T., DiRaimondo C.R., Stone W.J., Prelli F.C. (1986). Polymerization of intact β2-microglobulin in tissue causes amyloidosis in patients on chronic hemodialysis. Proc. Natl. Acad. Sci. U. S. A..

[8] Linke R.P., Hampl H., Lobeck H., Ritz E., Bommer J., Waldherr R. (1989). Lysine-specific cleavage of β2-microglobulin in amyloid deposits associated with hemodialysis. Kidney Int..

[9] Giorgetti S., Rossi A., Mangione P., Raimondi S., Marini S., Stoppini M. (2005). β_2_-Microglobulin isoforms display an heterogeneous affinity for type I collagen. Protein Sci..

[10] Benseny-Cases N., Karamanos T.K., Hoop C.L., Baum J., Radford S.E. (2019). Extracellular matrix components modulate different stages in β_2_-microglobulin amyloid formation. J. Biol. Chem..

[11] Hoop C.L., Zhu J., Bhattacharya S., Tobita C.A., Radford S.E., Baum J. (2020). Collagen I weakly interacts with the β-sheets of β2-microglobulin and enhances conformational exchange to induce amyloid formation. J. Am. Chem. Soc..

[12] Ohashi K., Kisilevsky R., Yanagishita M. (2002). Affinity binding of glycosaminoglycans with β2-microglobulin. Nephron.

[13] Myers S.L., Jones S., Jahn T.R., Morten I.J., Tennent G.A., Hewitt E.W. (2006). A systematic study of the effect of physiological factors on β2-microglobulin amyloid formation at neutral pH. Biochemistry.

[14] Valleix S., Gillmore J.D., Bridoux F., Mangione P.P., Dogan A., Nedelec B. (2012). Hereditary systemic amyloidosis due to Asp76Asn variant β2-microglobulin. N. Engl. J. Med..

[15] Mangione P.P., Esposito G., Relini A., Raimondi S., Porcari R., Giorgetti S. (2013). Structure, folding dynamics, and amyloidogenesis of D76N β2-microglobulin roles of shear flow, hydrophobic surfaces, and α-crystallin. J. Biol. Chem..

[16] Guthertz N., van der Kant R., Martinez R.M., Xu Y., Trinh C., Iorga B.I. (2022). The effect of mutation on an aggregation-prone protein: an *in vivo, in vitro*, and *in silico* analysis. Proc. Natl. Acad. Sci. U. S. A..

[17] Smith H.I., Guthertz N., Cawood E.E., Maya-Martinez R., Breeze A.L., Radford S.E. (2020). The role of the IT-state in D76N β2-microglobulin amyloid assembly: a crucial intermediate or an innocuous bystander?. J. Biol. Chem..

[18] Natalello A., Mangione P.P., Giorgetti S., Porcari R., Marchese L., Zorzoli I. (2016). Co-fibrillogenesis of wild-type and D76N β2-microglobulin: the crucial role of fibrillar seeds. J. Biol. Chem..

[19] Visconti L., Malagrinò F., Broggini L., De Luca C., Moda F., Gianni S. (2019). Investigating the molecular basis of the aggregation propensity of the pathological D76N mutant of β2-microglobulin: role of the denatured state. Int. J. Mol. Sci..

[20] Chong S.H., Hong J., Lim S., Cho S., Lee J., Ham S. (2015). Structural and thermodynamic characteristics of amyloidogenic intermediates of β2-microglobulin. Sci. Rep..

[21] Le Marchand T., de Rosa M., Salvi N., Sala B.M., Andreas L.B., Barbet-Massin E. (2018). Conformational dynamics in crystals reveal the molecular bases for D76N β2-microglobulin aggregation propensity. Nat. Commun..

[22] Eakin C.M., Berman A.J., Miranker A.D. (2006). A native to amyloidogenic transition regulated by a backbone trigger. Nat. Struct. Mol. Biol..

[23] Chiti F., Mangione P., Andreola A., Giorgetti S., Stefani M., Dobson C.M. (2001). Detection of two partially structured species in the folding process of the amyloidogenic protein β2-microglobulin. J. Mol. Biol..

[24] Kameda A., Hoshino M., Higurashi T., Takahashi S., Naiki H., Goto Y. (2005). Nuclear magnetic resonance characterization of the refolding intermediate of β_2_-microglobulin trapped by non-native prolyl peptide bond. J. Mol. Biol..

[25] Jahn T.R., Parker M.J., Homans S.W., Radford S.E. (2006). Amyloid formation under physiological conditions proceeds *via* a native-like folding intermediate. Nat. Struct. Mol. Biol..

[26] Eichner T., Kalverda A.P., Thompson G.S., Homans S.W., Radford S.E. (2011). Conformational Conversion during amyloid formation at atomic resolution. Mol. Cell.

[27] Karamanos T.K., Jackson M.P., Calabrese A.N., Goodchild S.C., Cawood E.E., Thompson G.S. (2019). Structural mapping of oligomeric intermediates in an amyloid assembly pathway. Elife.

[28] Loureiro R J.S., Vila-Viçosa D., Machuqueiro M., Shakhnovich E.I., Faísca P F.N. (2019). The early phase of β2m aggregation: an integrative computational study framed on the D76N mutant and the ΔN6 variant. Biomolecules.

[29] Karamanos T.K., Kalverda A.P., Thompson G.S., Radford S.E. (2014). Visualization of transient protein-protein interactions that promote or inhibit amyloid assembly. Mol. Cell.

[30] Kwan A.H., Mobli M., Gooley P.R., King G.F., Mackay J.P. (2011). Macromolecular NMR spectroscopy for the non-spectroscopist. FEBS J..

[31] Marion D. (2013). An introduction to biological NMR spectroscopy. Mol. Cell. Proteomics.

[32] Doherty C.P.A., Ulamec S.M., Maya-Martinez R., Good S.C., Makepeace J., Khan G.N. (2020). A short motif in the N-terminal region of α-synuclein is critical for both aggregation and function. Nat. Struct. Mol. Biol..

[33] Volkov A.N., Ubbink M., Van Nuland N.A.J. (2010). Mapping the encounter state of a transient protein complex by PRE NMR spectroscopy. J. Biomol. NMR.

[34] Ghose R. (2018). Protein NMR methods and protocols. Met. Mol. Biol..

[35] Walko M., Hewitt E., Radford S.E., Wilson A.J. (2019). Design and synthesis of cysteine-specific labels for photo-cross-linking studies. RSC Adv..

[36] Horne J.E., Walko M., Calabrese A.N., Levenstein M.A., Brockwell D.J., Kapur N. (2018). Rapid mapping of protein interactions using tag-transfer photocross-linkers. Angew. Chem. - Int. Ed..

[37] Iwata K., Matsuura T., Sakurai K., Nakagawa A., Goto Y. (2007). High-resolution crystal structure of β2-microglobulin formed at pH 7.0. J. Biochem..

[38] Karamanos T.K., Kalverda A.P., Radford S.E. (2022). Generating ensembles of dynamic misfolding proteins. Front. Neurosci..

[39] Misra P., Blancas-Mejia L.M., Ramirez-Alvarado M. (2019). Mechanistic insights into the early events in the aggregation of immunoglobulin light chains. Biochemistry.

[40] Halabelian L., Relini A., Barbiroli A., Penco A., Bolognesi M., Ricagno S. (2015). A covalent homodimer probing early oligomers along amyloid aggregation. Sci. Rep..

[41] Cawood E.E., Guthertz N., Ebo J.S., Karamanos T.K., Radford S.E., Wilson A.J. (2020). Modulation of amyloidogenic protein self-assembly using tethered small molecules. J. Am. Chem. Soc..

[42] Marcinko T.M., Dong J., LeBlanc R., Daborowski K.V., Vachet R.W. (2017). Small molecule-mediated inhibition of β2-microglobulin-based amyloid fibril formation. J. Biol. Chem..

[43] Nagaraj M., Najarzadeh Z., Pansieri J., Biverstål H., Musteikyte G., Smirnovas V. (2022). Chaperones mainly suppress primary nucleation during formation of functional amyloid required for bacterial biofilm formation. Chem. Sci..

[44] Kim N.H., Choi H., Shahzad Z.M., Ki H., Lee J., Chae H. (2022). Supramolecular assembly of protein building blocks: from folding to function. Nano Converg..

[45] Pieters B.J.G.E., Van Eldijk M.B., Nolte R.J.M., Mecinović J. (2016). Natural supramolecular protein assemblies. Chem. Soc. Rev..

[46] Chiesa G., Kiriakov S., Khalil A.S. (2020). Protein assembly systems in natural and synthetic biology. BMC Biol..

[47] Iadanza M.G., Jackson M.P., Hewitt E.W., Ranson N.A., Radford S.E. (2018). A new era for understanding amyloid structures and disease. Nat. Rev. Mol. Cell Biol..

[48] Otzen D., Riek R. (2019). Functional amyloids. Cold Spring Harb. Perspect. Biol..

[49] Rubel M.S., Fedotov S.A., Grizel A.V., Sopova J.V., Malikova O.A., Chernoff Y.O. (2020). Functional mammalian amyloids and amyloid-like proteins. Life.

[50] Seidler P.M., Murray K.A., Boyer D.R., Ge P., Sawaya M.R., Hu C.J. (2022). Structure-based discovery of small molecules that disaggregate Alzheimer’s disease tissue derived tau fibrils *in vitro*. Nat. Commun..

[51] Gao X., Carroni M., Nussbaum-Krammer C., Mogk A., Nillegoda N.B., Szlachcic A. (2015). Human Hsp70 disaggregase reverses Parkinson’s-linked α-synuclein amyloid fibrils. Mol. Cell.

[52] Chuang E., Hori A.M., Hesketh C.D., Shorter J. (2018). Amyloid assembly and disassembly. J. Cell Sci..

[53] Baldwin A.J., Knowles T.P., Tartaglia G.G., Fitzpatrick A.W., Devlin G.L., Shammas S.L. (2011). Metastability of native proteins and the phenomenon of amyloid formation. J. Am. Chem. Soc..

[54] Giorgetti S., Raimondi S., Pagano K., Relini A., Bucciantini M., Corazza A. (2011). Effect of tetracyclines on the dynamics of formation and destructuration of β2-microglobulin amyloid fibrils. J. Biol. Chem..

[55] Jha N.N., Kumar R., Panigrahi R., Navalkar A., Ghosh D., Sahay S. (2017). Comparison of α-synuclein fibril inhibition by four different amyloid inhibitors. ACS Chem. Neurosci..

[56] Morgan G.J., Yan N.L., Mortenson D.E., Rennella E., Blundon J.M., Gwin R.M. (2019). Stabilization of amyloidogenic immunoglobulin light chains by small molecules. Proc. Natl. Acad. Sci. U. S. A..

[57] Xu Y., Maya-Martinez R., Guthertz N., Heath G.R., Manfield I.W., Breeze A.L. (2022). Tuning the rate of aggregation of hIAPP into amyloid using small-molecule modulators of assembly. Nat. Commun..

[58] Garai K., Posey A.E., Li X., Buxbaum J.N., Pappu R.V. (2018). Inhibition of amyloid beta fibril formation by monomeric human transthyretin. Protein Sci..

[59] Nerelius C., Fitzen M., Johansson J. (2010). Amino acid sequence determinants and molecular chaperones in amyloid fibril formation. Biochem. Biophys. Res. Commun..

[60] Kasturirangan S., Li L., Emadi S., Boddapati S., Schulz P., Sierks M.R. (2012). Nanobody specific for oligomeric beta-amyloid stabilizes nontoxic form. Neurobiol. Aging.

[61] Raimondi S., Porcari R., Mangione P.P., Verona G., Marcoux J., Giorgetti S. (2017). A specific nanobody prevents amyloidogenesis of D76N β2- microglobulin *in vitro* and modifies its tissue distribution *in vivo*. Sci. Rep..

[62] Shvadchak V.V., Afitska K., Yushchenko D.A. (2018). Inhibition of α-synuclein amyloid fibril elongation by blocking fibril ends. Angew. Chem. Int. Ed..

[63] Kyriukha Y.A., Afitska K., Kurochka A.S., Sachan S., Galkin M., Yushchenko D.A. (2019). α-synuclein dimers as potent inhibitors of fibrillization. J. Med. Chem..

[64] Rezaei-Ghaleh N., Andreetto E., Yan L.M., Kapurniotu A., Zweckstetter M. (2011). Interaction between amyloid beta peptide and an aggregation blocker peptide mimicking islet amyloid polypeptide. PLoS One.

[65] Verhelle A., Nair N., Everaert I., Van Overbeke W., Supply L., Zwaenepoel O. (2017). AAV9 delivered bispecific nanobody attenuates amyloid burden in the gelsolin amyloidosis mouse model. Hum. Mol. Genet..

[66] Bulawa C.E., Connelly S., Devit M., Wang L., Weigel C., Fleming J.A. (2012). Tafamidis, a potent and selective transthyretin kinetic stabilizer that inhibits the amyloid cascade. Proc. Natl. Acad. Sci. U. S. A..

[67] Bellotti V., Stoppini M., Mangione P., Sunde M., Robinson C., Asti L. (1998). β_2_-Microglobulin can be refolded into a native state from *ex vivo* amyloid fibrils. Eur. J. Biochem..

[68] Tonge P.J. (2018). Drug-target kinetics in drug discovery. ACS Chem. Neurosci..

[69] Colombo M., De Rosa M., Bellotti V., Ricagno S., Bolognesi M. (2012). A recurrent D-strand association interface is observed in β2-microglobulin oligomers. FEBS J..

[70] Rennella E., Cutuil T., Schanda P., Ayala I., Gabel F., Forge V. (2013). Oligomeric states along the folding pathways of β2-microglobulin: kinetics, thermodynamics, and structure. J. Mol. Biol..

[71] Kad N.M., Thomson N.H., Smith D.P., Smith D.A., Radford S.E. (2001). β_2_-microglobulin and its deamidated variant, N17D form amyloid fibrils with a range of morphologies *in vitro*. J. Mol. Biol..

[72] Maciejewski M.W., Schuyler A.D., Gryk M.R., Moraru I.I., Romero P.R., Ulrich E.L. (2017). NMRbox: a resource for biomolecular NMR computation. Biophys. J..

[73] Delaglio F., Grzesiek S., Vuister G.W., Zhu G., Pfeifer J., Bax A. (1995). NMRPipe: a multidimensional spectral processing system based on UNIX pipes. J. Biomol. NMR.

[74] Skinner S.P., Fogh R.H., Boucher W., Ragan T.J., Mureddu L.G., Vuister G.W. (2016). CcpNmr AnalysisAssign: a flexible platform for integrated NMR analysis. J. Biomol. NMR.

[75] Niklasson M., Andresen C., Helander S., Roth M.G., Zimdahl Kahlin A., Lindqvist Appell M. (2015). Robust and convenient analysis of protein thermal and chemical stability. Protein Sci..

[76] Hagelueken G., Ward R., Naismith J.H., Schiemann O. (2012). MtsslWizard: *in silico* spin-labeling and generation of distance distributions in PyMOL. Appl. Magn. Reson..

[77] van Zundert G.C.P., Rodrigues J.P.G.L.M., Trellet M., Schmitz C., Kastritis P.L., Karaca E. (2016). The HADDOCK2.2 web server: user-friendly integrative modeling of biomolecular complexes. J. Mol. Biol..

