# Supplementary material for: Dimers of D76N-β2-microglobulin display potent antiamyloid aggregation activity
Source: J Biol Chem. 2022 Oct 31;298(12):102659. doi: 10.1016/j.jbc.2022.102659 (PMC9712992; doi:10.1016/j.jbc.2022.102659)
Supplement: Supporting information [file mmc1.docx]

Dimers of D76N-β_2_-microglobulin display potent anti-amyloid aggregation activity

**Roberto Maya-Martinez^1^, Yong Xu^1^, Nicolas Guthertz^1^, Martin Walko^2^, Theodoros K. Karamanos^1^, Frank Sobott^1^, Alexander L. Breeze^1^, Sheena E. Radford^1*^**

^1^From the Astbury Centre for Structural Molecular Biology, School of Molecular and Cellular Biology, Faculty of Biological Sciences, University of Leeds, Leeds LS2 9JT, UK

^2^Astbury Centre for Structural Molecular Biology, School of Chemistry, University of Leeds, Leeds LS2 9JT, UK

**Running title:** Inhibitory dimers of D76N-β_2_m aggregation

*To whom correspondence should be addressed: Sheena E. Radford, Astbury Centre for Structural Molecular Biology and School of Molecular and Cellular Biology, University of Leeds, Leeds LS2 9JT Telephone: +44 113 343 3170; email: s.e.radford@leeds.ac.uk.

**Supplementary Figures**

**Suppelementary Figure S1**

**Figure S1. Melting temperature of D76N-Cys-MTSL-β_2_m variants.** Thermal denaturation using far UV CD was used to determine the T_m_ of each variant labelled with MTSL. All samples (20 µM) were dissolved in 25 mM sodium phosphate, 115mM NaCl, pH 6.2. The discontinuous black line highlights that each protein is natively folded at the temperature at which the NMR PRE experiments were acquired (25 °C). The T_m_ values for each protein are 53.2 ± 0.2 °C (D76N-β_2_m), 42.9 ± 0.4 °C (D76N-20C-MTSL), 43.3 ± 0.2 °C (D76N-33C-MTSL), 44.1 ± 0.2 °C (D76N-57-MTSL), 38.1 ± 0.3 °C (D76N-88C-MTSL). The error is the error to the fit.

**Supplementary Figure S2**


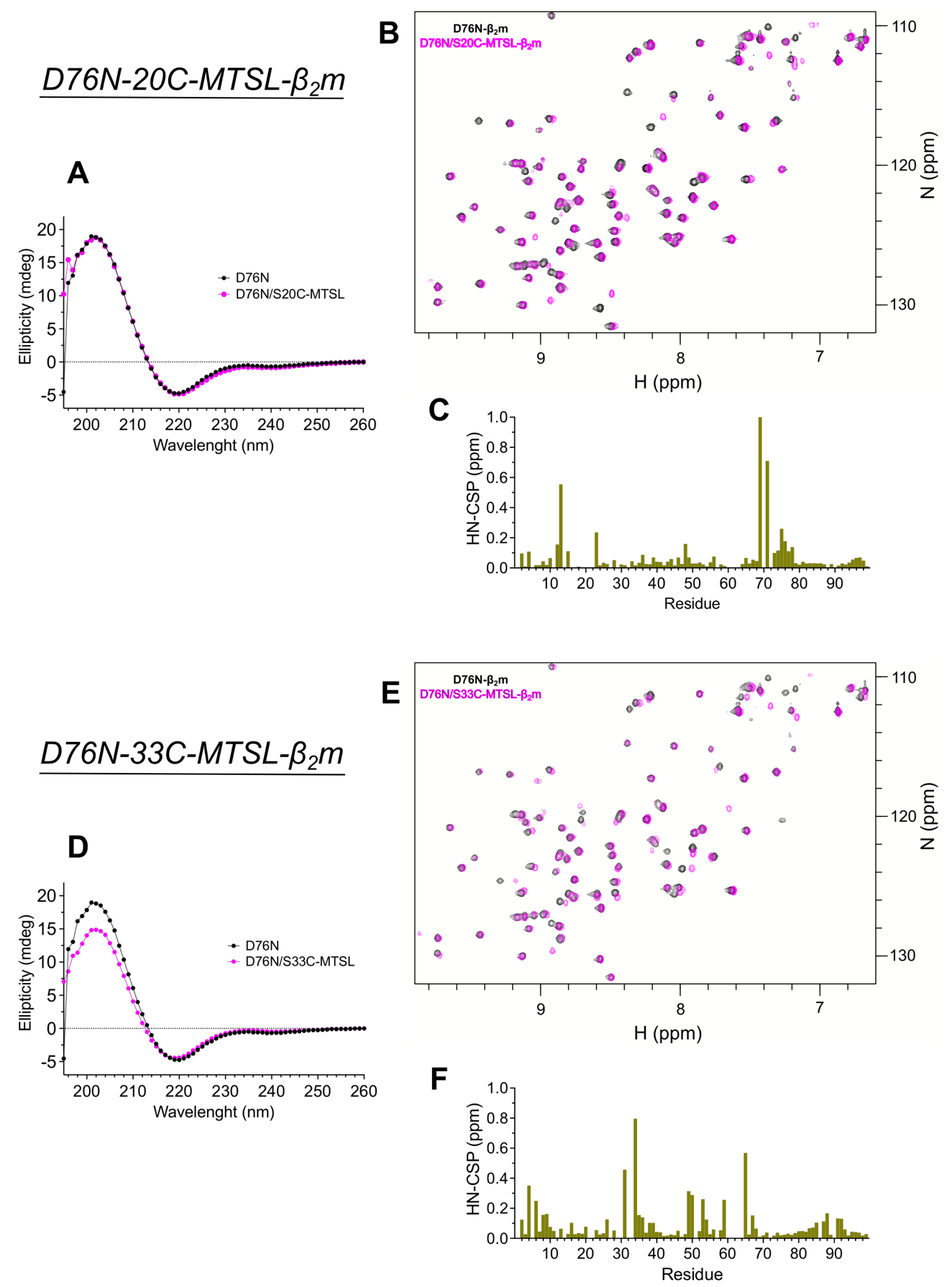


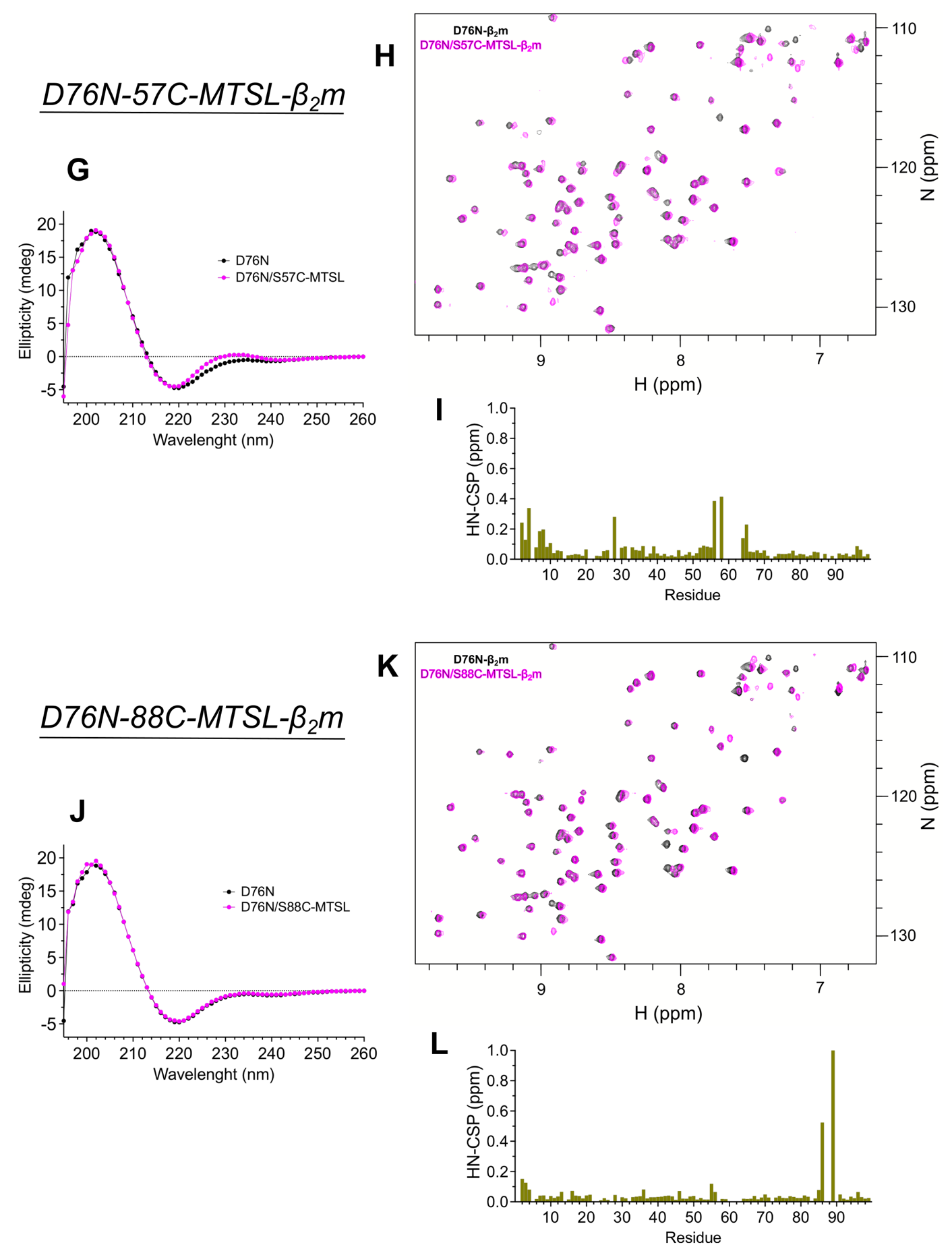


**Figure S2. Secondary and tertiary structure characterization of D76N-Cys-MTSL variants at 25 °C and pH 6.2.** The effect of -cysteine and -MTSL insertion on D76N-β_2_m at residues 20, 33, 57 and 88 was evaluated using far UV CD (A, D, G and J, respectively), ^HN^HSQC (diamagnetic sample) (B, E, H and K, respectively) and analysis of chemical shift perturbations compared with unmodified D76N-β_2_m (C, F, I and K, respectively). All experiments were performed in 25 mM sodium phosphate, 115 mM NaCl, 25 ℃ and pH 6.2.

**Supplementary Figure S3**


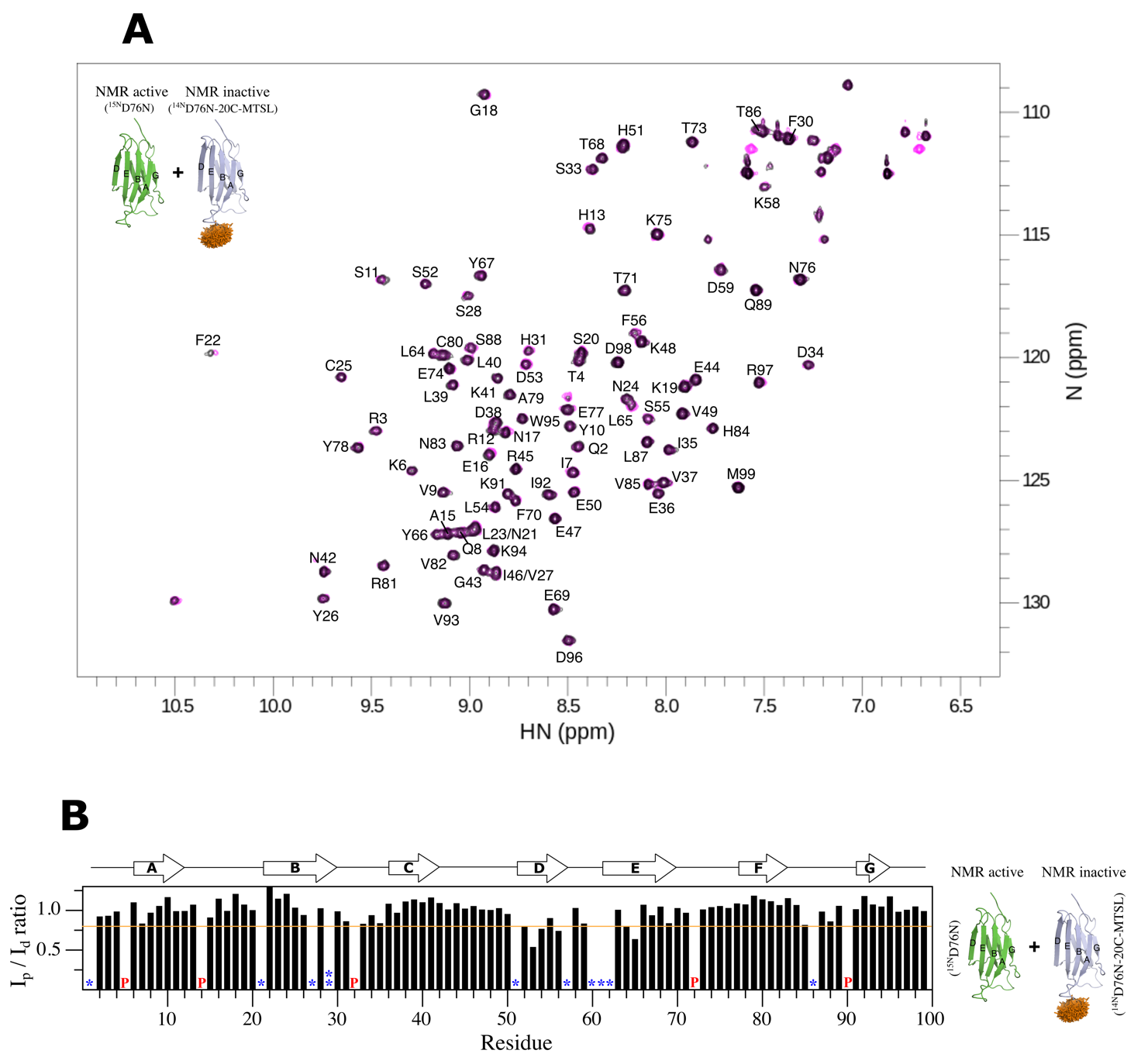


**Figure S3. Inter-molecular NMR PRE data for D76N/20C-MTSL-β_2_m. (A)** 2D ^HN^HSQC spectra of ^15^N-D76N-β_2_m mixed with equimolar (100 μM) ^14^N-D76N-β_2_m/20C-MTSL under diamagnetic (I_d_, pink cross-peaks) and paramagnetic (I_p_, black cross-peaks) conditions. The structure (inset) shows the position where MTSL is inserted in ^14^N-D76N-β_2_m/20C-MTSL (represented in orange). **(B)** Cross-peak intensities extracted from spectra in **(A)**. Data are plotted as the I_p_/I_d_ ratio. The horizontal orange line represents the significance threshold applied to the data (0.8). Single and double asterisks refer to residues not assigned or HN-line broadening under diamagnetic conditions, respectively. P represents prolines. The secondary structure of D76N-β_2_m (4FXL (14)) is shown above the graph in (**B**). Structural representation of the inter-molecular PRE-reaction is shown inset in (A) and to the right side of (B). The position of MTSL probe is shown in orange as an ensemble of conformations in ^14N^D76N-β_2_m. A schematic showing the proteins mixed in each experiment is inset in each graph: green ^15^N-D76N-β_2_m, grey ^14^N-D76N-β_2_m Cys variants labelled with MTSL, MTSL (orange stick). **Supplementary Figure S4**


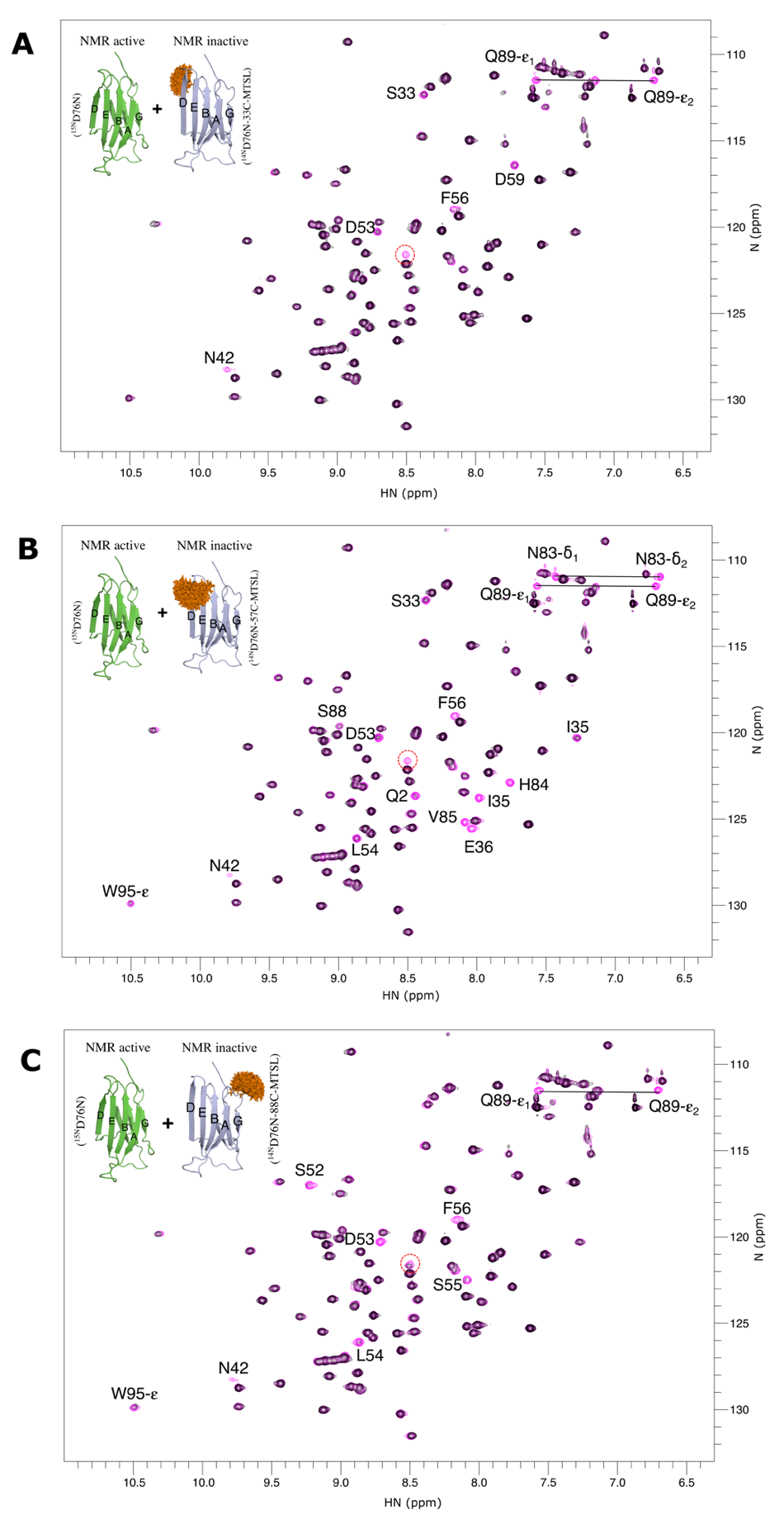


**Figure S4. Inter-molecular NMR PRE data for D76N/C33-, /C57- or /C88-β_2_m mixed with ^15N^D76N-β_2_m.** 2D  ^HN^HSQC spectra of ^15^N-D76N-β_2_m mixed with equimolar (100 μM) **(A)** D76N/33C-MTSL β_2_m; (**B)** D76N/57C-MTSL β_2_m; or (**C)** D76N/88C-MTSL β_2_m (each ^14^N). Diamagnetic and paramagnetic conditions are shown in pink and black, respectively. All spectra were recorded at 25 ℃ using a 750 MHz spectrometer. Residues that experience the greatest PRE effect are highlighted with their respective residue labels. One residue with missing assignment (no ^13^C resonance connectivities are observed in 3D spectra) and a strong PRE is highlighted with a dotted red circle. A schematic of the proteins mixed in the inter-molecular PRE-experiment is shown inset in each plot: green ^15^N-D76N-β_2_m, grey ^14^N-D76N-β_2_m Cys variants labelled with MTSL, MTSL (orange stick).

**Supplementary Figure S5**


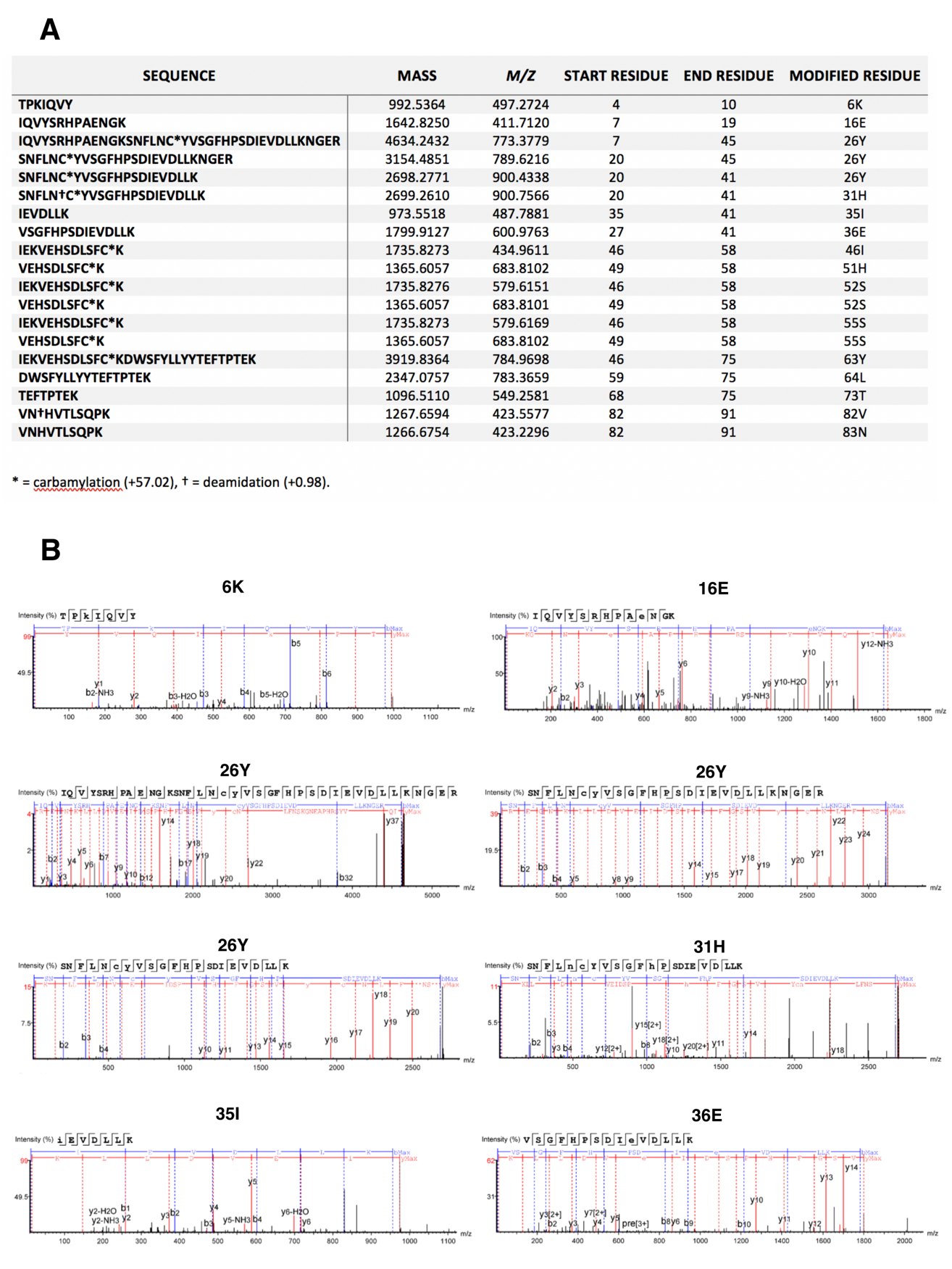


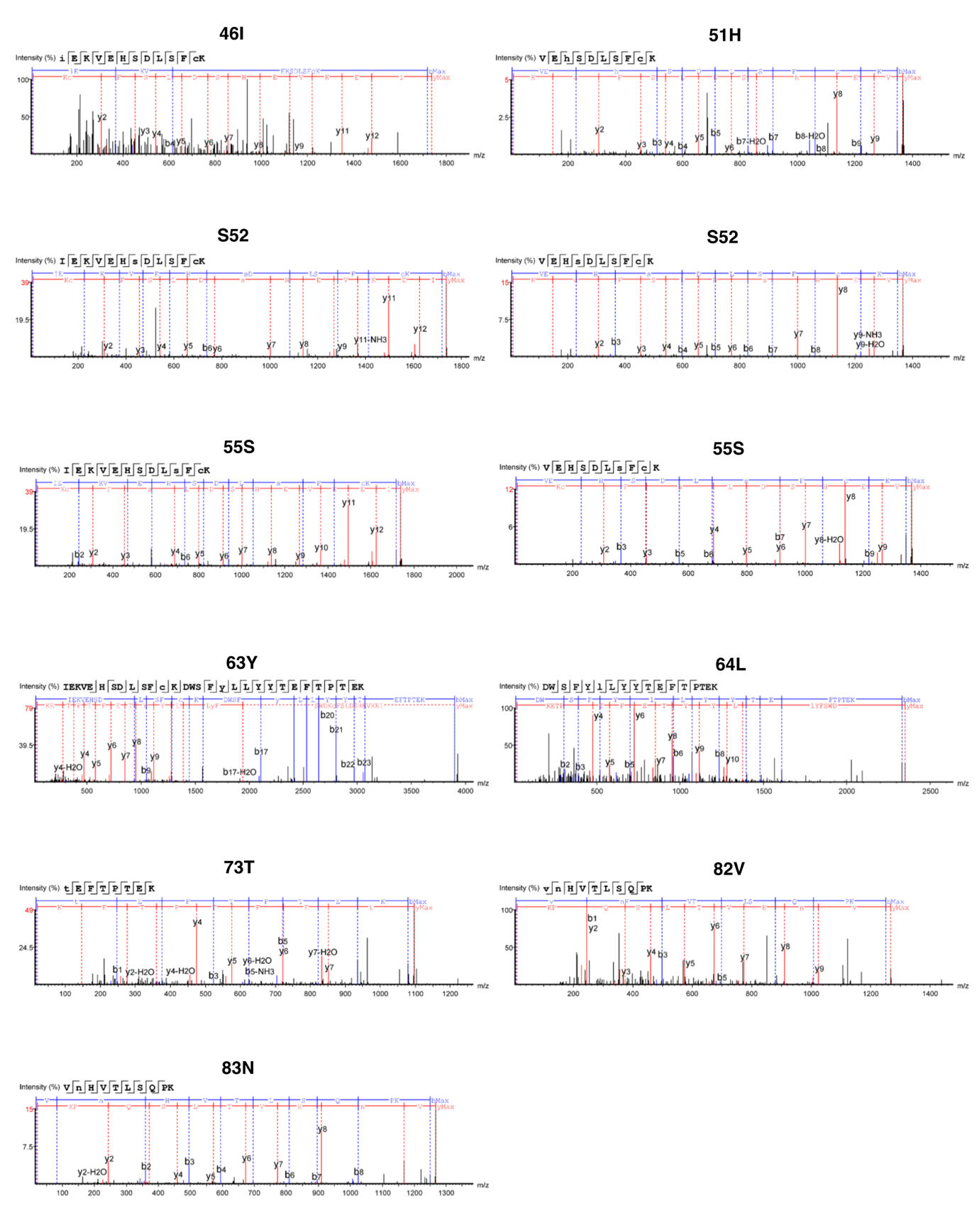
**Figure S5. Identification of the cross-links in the D76N-β_2_m-57C-diazirine dimer.** LC-MS traces of the modified peptides obtained using tag -transfer for D76N-β_2_m-57C-diazirine dimers. Peptides are listed, along with the modification sites in **Supplementary Table S1**.

**Supplementary Figure S6**

**
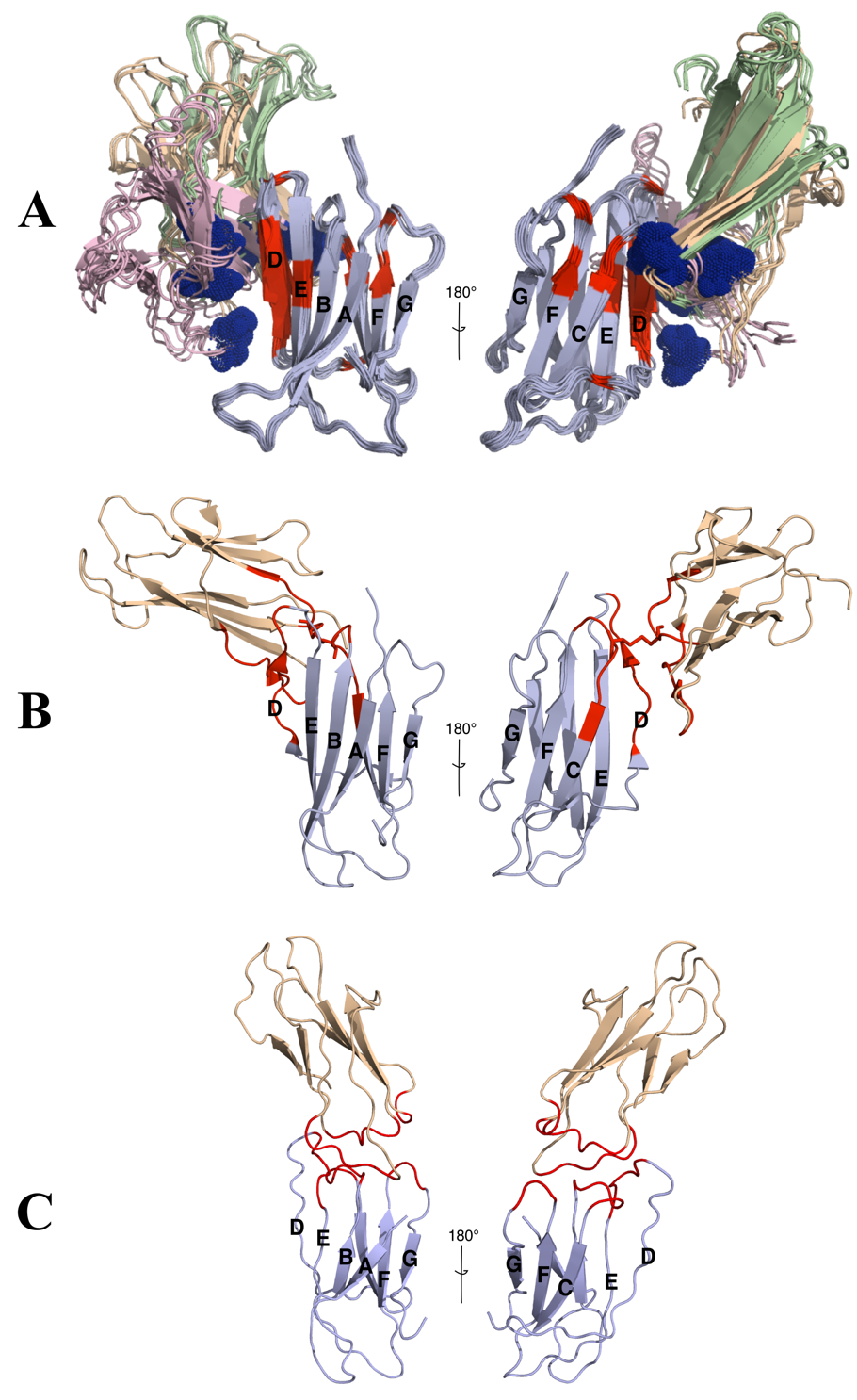
**

**Supplementary Fig. S6.** **Comparison of β_2_m dimers reported in the literature**. **(A)** D76N-β_2_m cross-linked dimer reported in this work, monomers are arranged in a ‘top-to-side’ orientation (as shown in figure 4 of the main text). **(B)** WT-β_2_m Cys33 covalently linked dimer (40), adopting top – side orientation similar to dimers in **(A)**. **(C)** ΔN6-β_2_m non-covalent dimer (26), forming a stable conformer dominated by ‘top-to-top’ interaction. The monomer – monomer interface is shown in each case in red.

**Supplementary Figure S7**


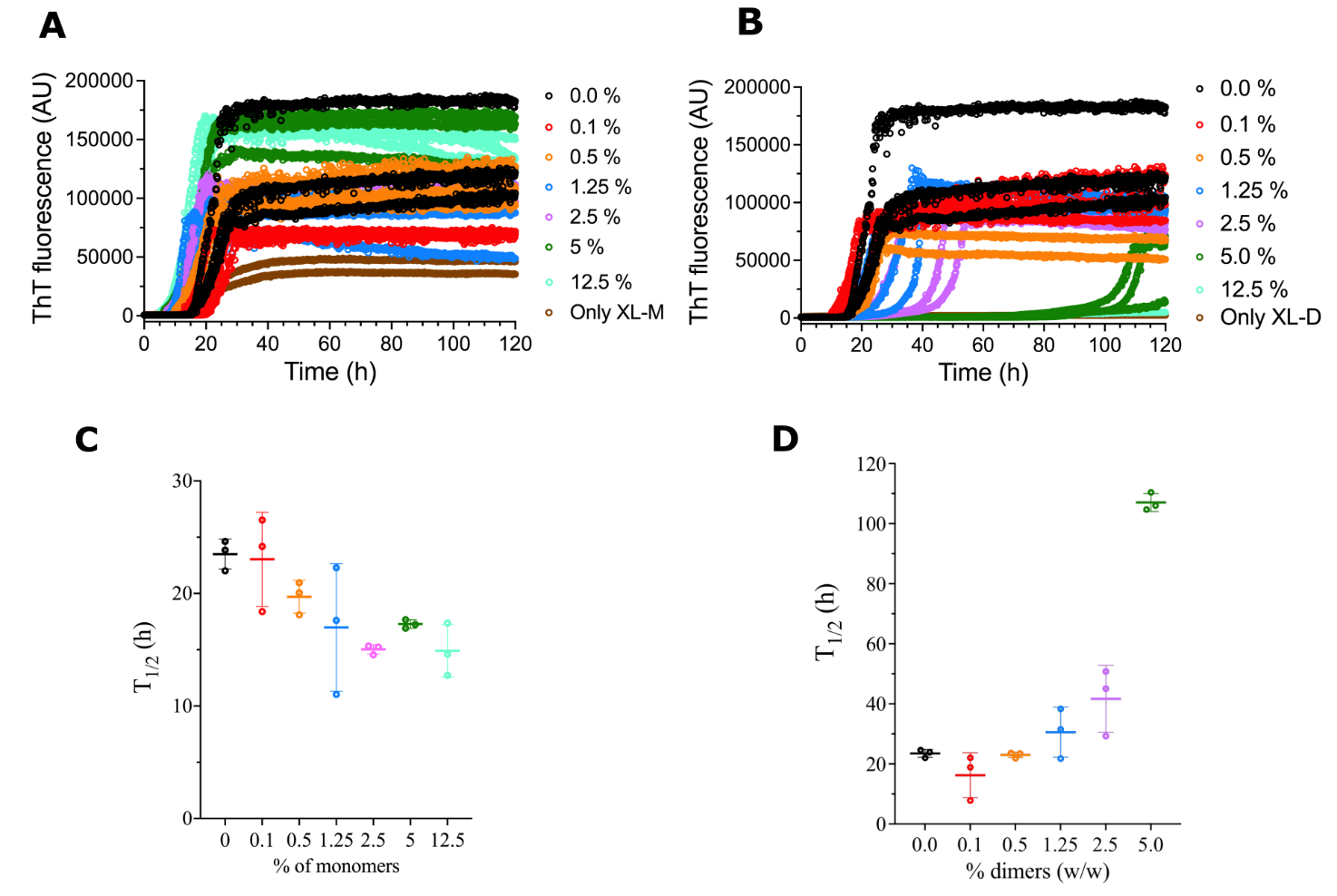


**Figure S7. Fibrillation kinetics of D76N-β_2_m monomers in presence of increasing concentrations of D76N/57C-β_2_m cross-linked monomers or dimers. (A)** ThT fibrillation curves of D76N-β_2_m monomers (20 μM) in the presence of different percentages of cross-linked monomers, spanning 0 – 12.5% (*w/w*) (the key on the right side shows the color code for each concentration used). Sample containing only cross-linked monomers (XL-M) is shown in brown. (**B)** As **(A)**, but for cross-linked dimers (XL-D). T_50_ values for D76N-β_2_m fibrillation vs % of cross-linked monomers or dimers are shown in **(C)** and **(D),** respectively. All experiments were performed in 25mM sodium phosphate, 115mM NaCl, pH 6.2, at 37 ℃ with 600 rpm shaking. Note that while significant retardation in amyloid formation occurs in the presence of cross-linked dimers, mild acceleration occurs in the presence of cross-linked monomers, possibly because the total protein concentration is increased, although a direct effect of cross-linked monomers increasing the rate of amyloid formation cannot be ruled out.

**Supplementary Figure S8**


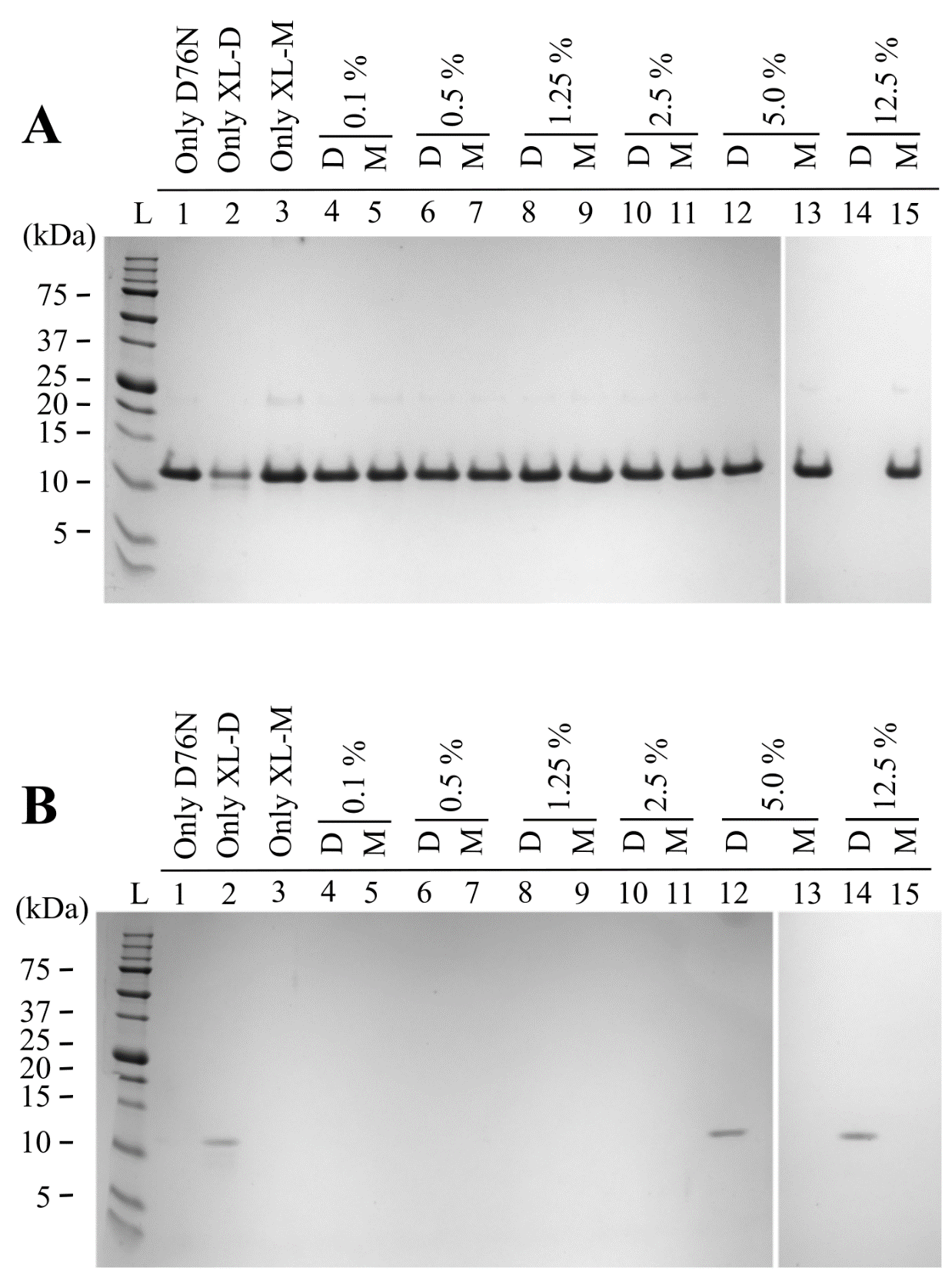


**Figure S8. SDS-PAGE analysis of insoluble and soluble fractions after 120 h of incubation of D76N-β_2_m in presence of varying percentages of cross-linked monomers or dimers. (A)** Insoluble fractions (pelleted after 10 min centrifugation at 23,000 *g*) (Experimental Procedures). A reduction in, or lack of, insoluble material was only observed in samples containing either 100 % dimers (lane 2) or supplemented with 12.5 % (w/w) of cross-linked dimers (lane 14). (**B)** Soluble fractions. Soluble material was only observed in those conditions that contain: only dimers (lane 2), or supplemented with 5% (lane 12) or 12.5% (lane 14) dimers, respectively. L refers to protein ladder (kDa). M and D denotes the addition of monomeric or dimeric cross-linked species, respectively. D76N-β_2_m has a molecular weight of 11.86 kDa. Note that cross-linked species migrate as a monomer in these reducing SDS gels. The band intensities in **(A)** and **(B)** cannot be directly compared as different amounts of protein were loaded onto each gel.

**Supplementary Figure S9**


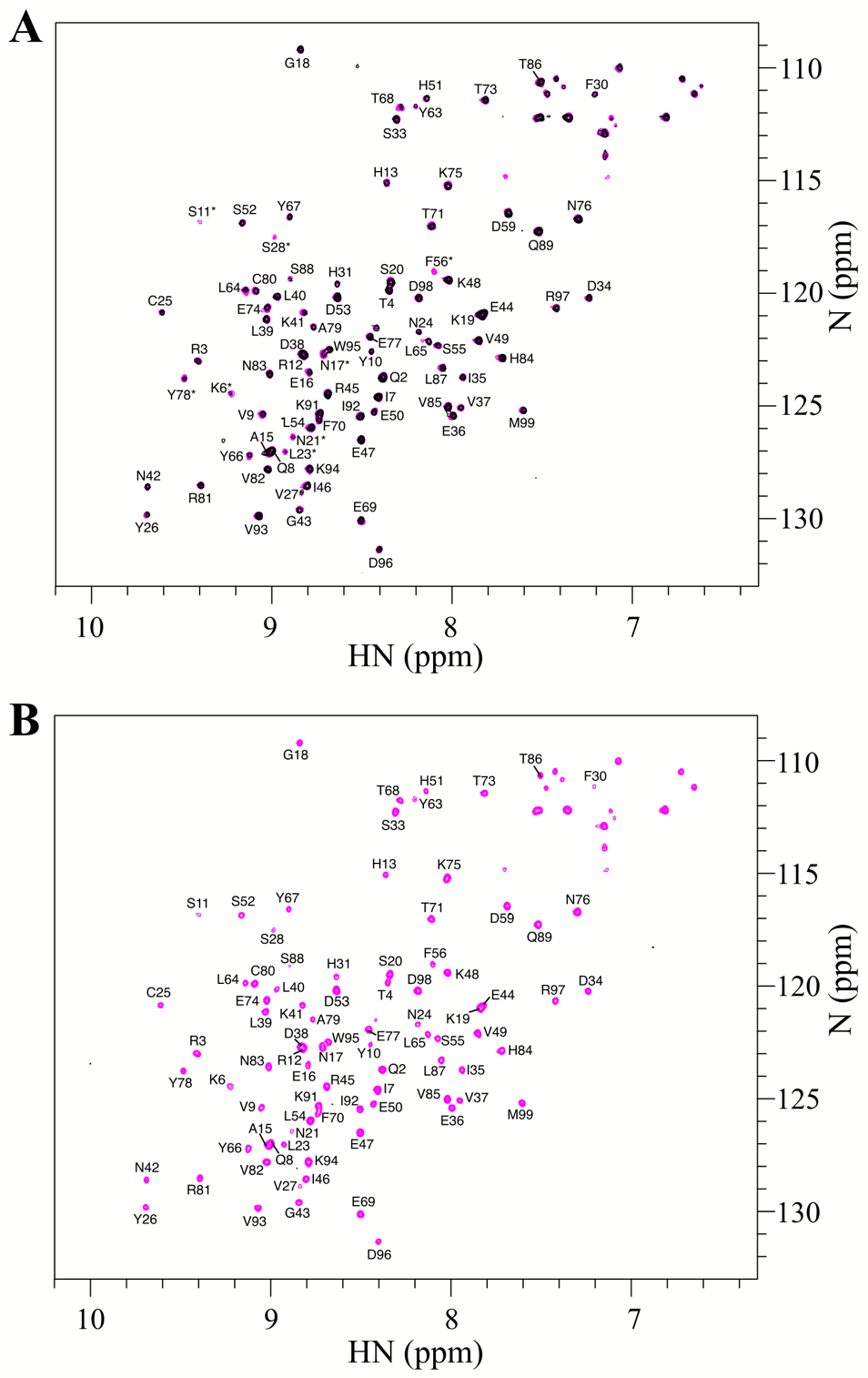


**Figure S9. Conformation of D76N-β_2_m after 38 h of fibrillation with/without D76N-β_2_m cross-linked dimers. (A**) Superposition of 2D ^HN^SOFAST/HSQC-NMR spectra of D76N-β_2_m in the presence of 10 % (*w/w*) D76N/57C cross-linked dimers at time 0 h (pink cross peaks) and after incubation for 38 h (black cross peaks). The spectra show complete inhibition of D76N-β_2_m aggregation and show that the product of the incubation is native-like monomers. (**B)**, as **(A)** but in the absence of cross-linked dimers. The absence of black cross peaks after 38 h incubation in **(B)** demonstrates the complete depletion of monomer into NMR-invisible aggregates under these conditions. All NMR spectra were acquired at 37 °C on a 950 MHz NMR spectrometer.

**Supplementary Figure S10**

**(A) (B)**


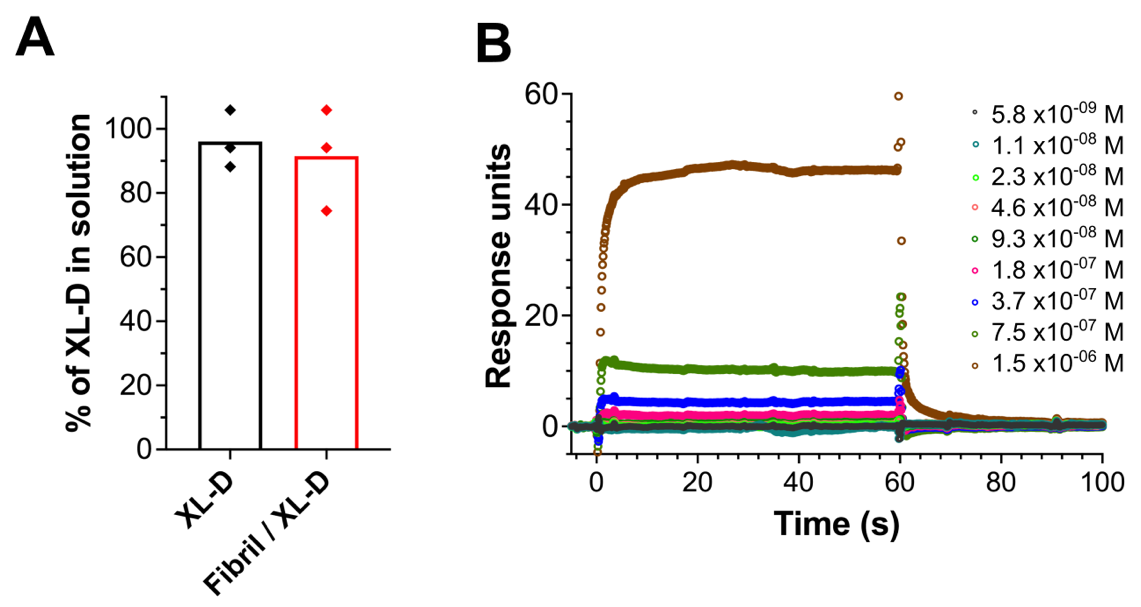


**Figure S10**. **Interactions of cross-linked dimers (XL-D) with D76N-β_2_m fibrils and monomers. (A)** Quantification of XL-D in solution after pelleting with/without D76N-β_2_m pre-formed fibrils. Black bar: XL-D remaining in solution after incubation without fibrils; red bar XL-D remaining in solution after incubation with fibrils. Each experiment was performed in triplicate. Samples were in 25 mM sodium phosphate, 115 mM NaCl, pH 6.2 (20 μM fibril (monomer equivalent concentration) and 2 μM XL-D). **(B)** SPR analysis of the binding of XL-D (concentrations ranging from 5.8 nM to 1.5 μM) with immobilized monomers of D76N-β_2_m on the streptavidin chip (see Experimental Procedures). All SPR experiments were performed in 25 mM sodium phosphate, 115 mM NaCl at pH 6.2. The results show no stable interaction of XL-D with pre-formed fibrils (**A**) and clear evidence for an interaction between D76N-β_2_m monomers and XL-D (**B**).

**Supplementary Tables and Table Legends**

**Supplementary Table S1**


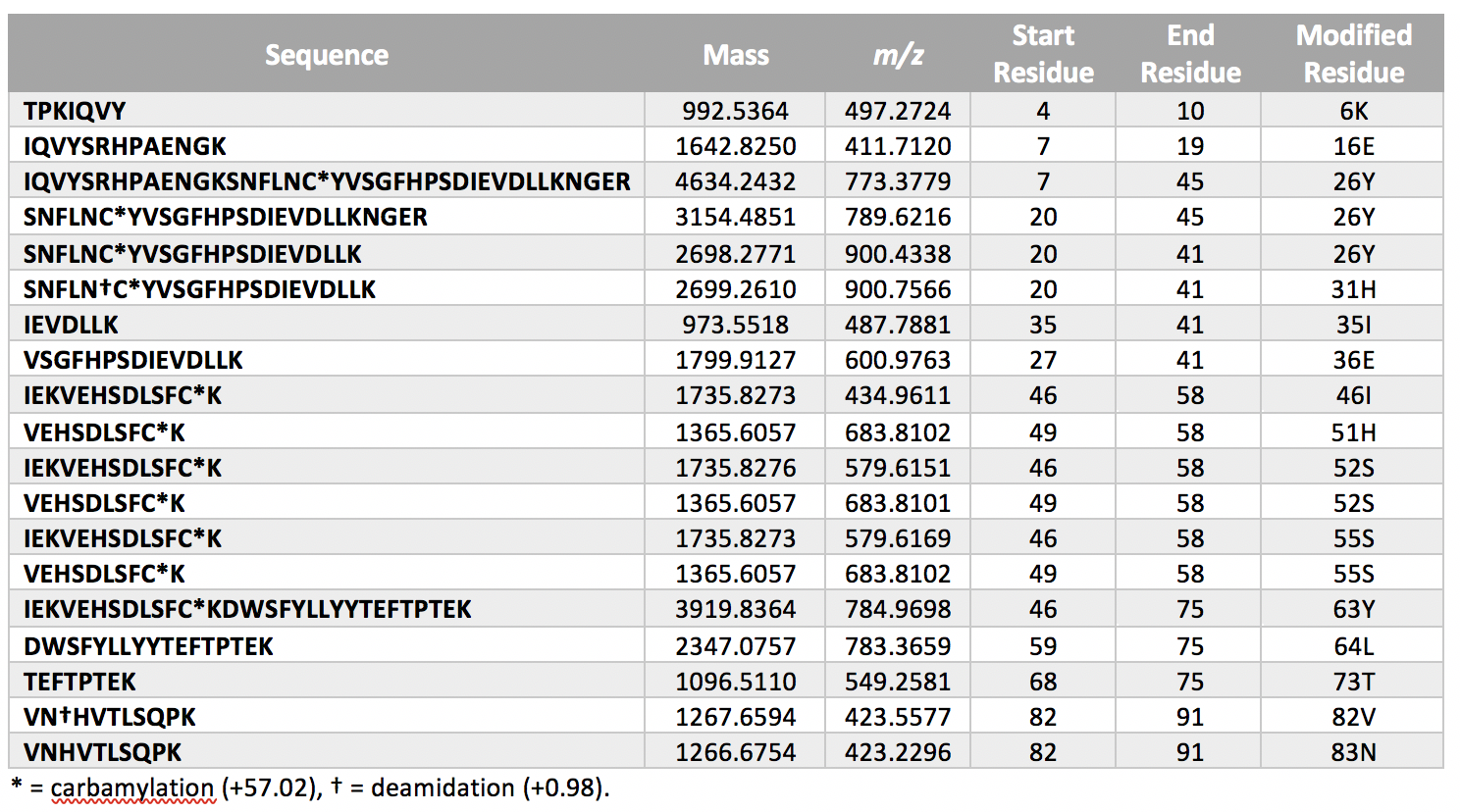


**Table S1. MTS-diazirine tag-transfer identification of the inter-protein interface in cross-linked D76N-β_2_m dimers.** List of D76N-β_2_m peptides identified by LC-MS that are modified by a covalently attached diazirine group. Sequence, mass, mass/charge distribution, residue coverage and modified residue of the identified peptides are shown. Carbamylation and deamidation modifications to some of the peptides are acquired during the purification step.

**Supplementary Table S2**


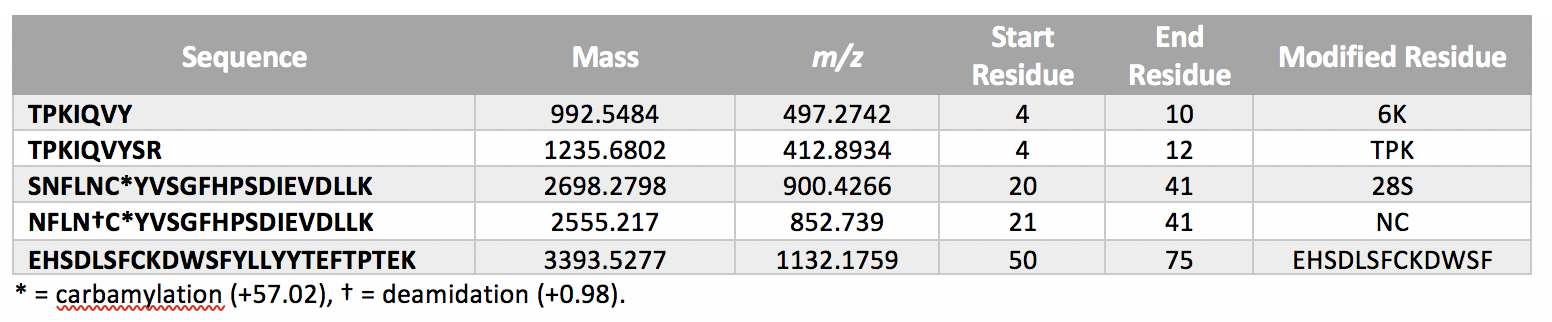


**Table S2. MTS-diazirine tag-transfer identification of intra-molecularly cross-linked D76N-β_2_m monomers by LC-MS.** List of peptides that are modified by a covalently attached the diazirine group from the tag transfer process. Sequence, mass, mass/change distribution, residue coverage and modified residue of the identified peptides are shown. Carbamylation and deamidation modifications to some of the peptides are acquired during the purification step.

**Supplementary Table S3**

| **Probe position** | **PRE (NMR) threshold and constraints** | | | **Cross-linking (Mass-Spec)** |
| --- | --- | --- | --- | --- |
|  | **Disappear** | **0.05 < 0.2** | **0.2 < 0.6** |  |
| S33C | 0 | 2 | 9 | ------ |
| S57C | 0 | 5 | 15 | 9 |
| S88C | 5 | ------ | 8 | ------ |

**Table S3. PRE-NMRs and cross-linking constraints used to create the dimeric D76N-β_2_m model.** With respect to PRE-NMR constraints, only data from S33C, S57C and S88C probes were used for this calculation. A probe-HN distance of ≤ 12 Å was assumed for residues that disappear and have PRE below < 0.2 Å, and ≤ 18 Å for all residues that have PRE values in the range of 0.2 < 0.6. For cross-linking, constraints from the S57C probe were used with a fixed distance of 12 Å for each cross-linked site. PRE-NMR restraints from S20C were not included as no significant PRES were observed from this site.

**Supplementary Table S4**

| *Parameter* | *Cluster # 1* | *Cluster # 2* | *Cluster #3* |
| --- | --- | --- | --- |
| *Haddock score* | 64.0 ± 5.8 | 19.7 ± 16.9 | 96.7 ± 9.7 |
| *Cluster size* | 145/200 | 38/200 | 5/200 |
| *RMSD from the overall lowest energy structure* | 13.0 ± 0.1 | 15.8 ± 0.1 | 12.3 ± 0.1 |
| *Van der Waals energy* | -31.4 ± 3.3 | -40.1 ± 1.1 | -36.8 ± 3.5 |
| *Electrostatic energy* | -197 ± 26.5 | -214.8 ± 47.5 | -79.7 ± 21.2 |
| *Desolvation energy* | -15.1 ± 1.4 | -18.7 ± 1.1 | -14.6 ± 3.8 |
| *Buried surface area* | 1102.5 ± 43.4 | 1281.1 ± 43.6 | 1131.8 ± 50.9 |
| *Z-score* | 0.1 | -1.3 | 1.2 |

**Table S4. HADDOCK results of D76N-β_2_m dimer model calculations.** Quality control parameters of the three lowest energetic clusters obtained during the docking calculation are shown. Cluster size is shown as representative number of structures per cluster over a total of 200 requested during the calculation.

**Supplementary Table S5**

| **Probe position** | **Satisfied constraints** | | | |
| --- | --- | --- | --- | --- |
|  | **S33C** | **S57C** | **S88C** | **Mass-Spec (S57C)** |
| Model 1 | 9 / 11 | 5 / 20 | 4 / 13 | 2 / 9 |
| Model 2 | 9 / 11 | 14 / 20 | 0 / 13 | 9 / 9 |
| Model 3 | 9 / 11 | 14 / 20 | 10 / 13 | 9 / 9 |

**Table S5. Summary of satisfied constraints of the generated D76N-β_2_m model.** The list of constraints per probe that are satisfied in D76N-β_2_m models generated are shown. Constraints are reported based on the total number per probe.
